# Supplementary material for: Tension-sensitive LINC-RhoA signaling prevents chromatin bridge breakage in cytokinesis
Source: EMBO J. 2025 Sep 9;44(20):5834–59. doi: 10.1038/s44318-025-00565-3 (PMC12528419; doi:10.1038/s44318-025-00565-3)
Supplement: Supplementary file 1 — Appendix [file 44318_2025_565_MOESM1_ESM.pdf]

Appendix for: "Tension-sensitive LINC-RhoA signaling prevents chromatin bridge breakage in cytokinesis"

Table of Contents

**Appendix Figure**	**Figure titles**	**Page**
S1	Expression of siRNA-resistant RhoA:GFP rescues actin patches and prevents chromatin bridge breakage in cytokinesis	3
S2	Inhibition of LIMK impairs actin patches and correlates with chromatin bridge breakage in cytokinesis	5
S3	Expression of siRNA-resistant Citrine:PDZ rescues actin patches and prevents chromatin bridge breakage in cytokinesis	7
S4	Expression of constitutively active GFP:FAK-Y397E rescues actin patches and prevents chromatin bridge breakage in RhoA-deficient cells	9
S5	Expression of constitutively active GFP:RhoA-G14V does not rescue actin patches in Src-deficient cells	11
S6	Expression of dominant-negative (dn) KASH domain impairs actin patches and correlates with increased frequency of broken chromatin bridges in cytokinesis	13
S7	Depletion of Nesprin-2 correlates with chromatin bridge breakage in living cells	15
S8	Nesprin-2 Spectrin Repeats 31-37 associate with the RH and DH/PH domains of PDZ RhoGEF	17
S9	Expression of mini-Nesprin-2 CH* or CH*:DHPH proteins diminishes nuclear chromatin shape-deformation in cytokinesis with intact chromatin bridges	20

Appendix Figure S1

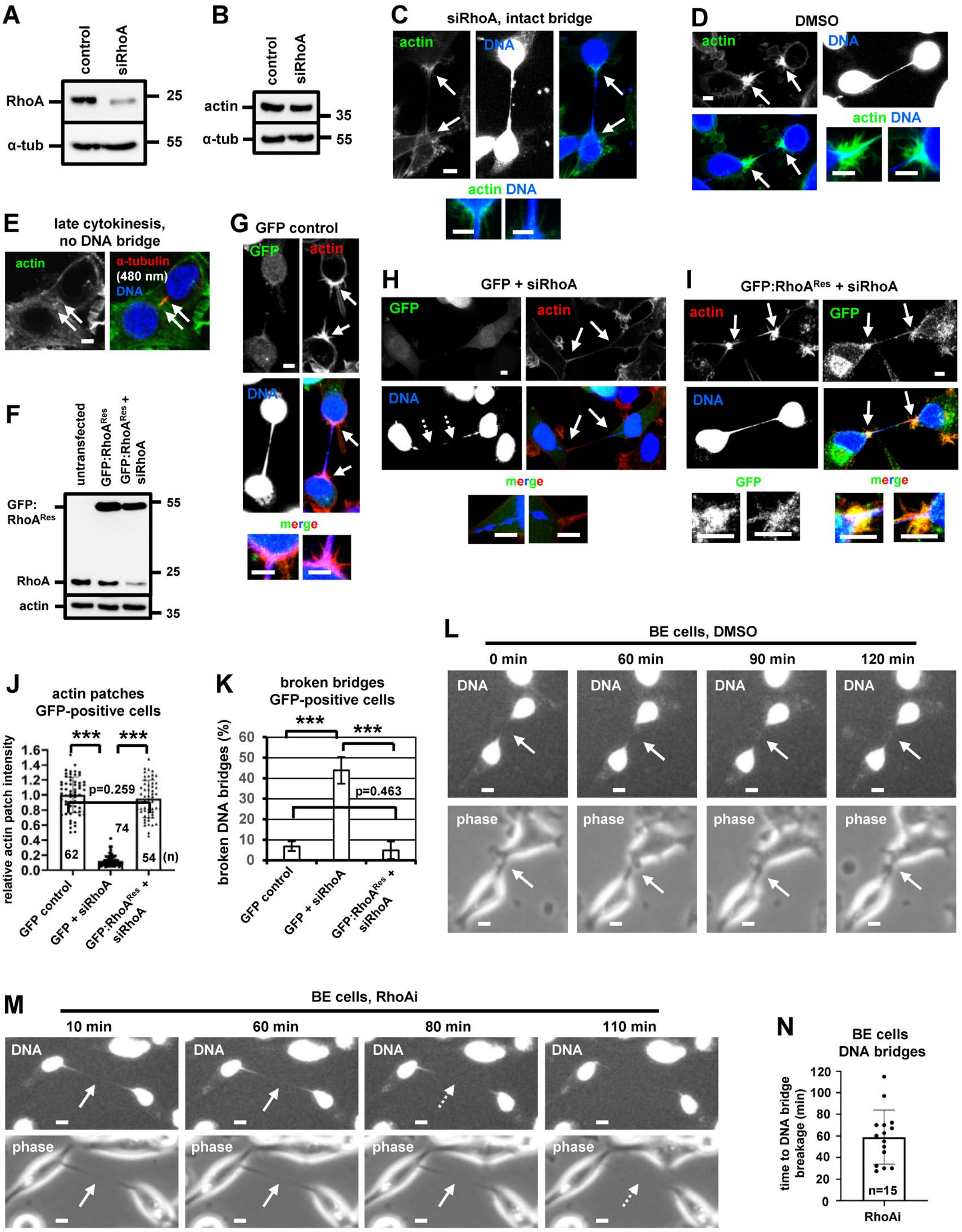

Appendix Figure S1. Expression of siRNA-resistant RhoA:GFP rescues actin patches and prevents chromatin bridge breakage in cytokinesis. (A, B) Western blot analysis of total RhoA, α-tubulin (α-tub) and actin in the absence (control) or presence of RhoA siRNA (siRhoA). **(C, D)** Actin patches and chromatin bridges in BE cells treated with siRhoA or 50 µM DMSO for 30 min. **(E)** Actin staining in cells in late cytokinesis without chromatin bridges connected by a late midbody. Tubulin values indicate midbody thickness. **(F)** Western blot analysis of total RhoA and actin in cells transfected with siRNA-resistant RhoA:GFP (GFP:RhoA^{Res}). **(G-I)** Actin patches and chromatin bridges in cells expressing GFP or GFP:RhoA^{Res} in the absence or presence of siRhoA. **(J)** Actin patches intensity in GFP-positive cells. Mean ± SD from n cells from two independent experiments. Values in GFP control were set to 1. Numbers below/next to each bar indicate n. ****P*= 1.07E-61 (GFP control vs GFP+siRhoA), 2.92E-54 (GFP+siRhoA vs GFP:RhoA^{Res}+siRhoA) by ANOVA and Student's t test. **(K)** Percentage of broken DNA bridges. Mean ± SD from three independent experiments (n= 87, 62, 41). ****P*= 6.66E-05 (GFP control vs GFP+siRhoA), 0.00062 (GFP+siRhoA vs GFP:RhoA^{Res}+siRhoA) by ANOVA and Student's t test. **(L, M)** BE cells labelled with Biotracker DNA dye were analyzed by live-cell imaging in the presence of 50 µM Y16 (RhoAi) or 50 µM DMSO. Time is from the detection of DNA bridges. **(N)** Time to DNA bridge breakage. Mean ± SD from n cells. Intact arrows indicate actin patches, intact DNA bridges or intercellular canals. Broken arrows indicate broken DNA bridges or intercellular canals. Insets show high magnifications of the canal bases. Bars, 5 µm.

Appendix Figure S2

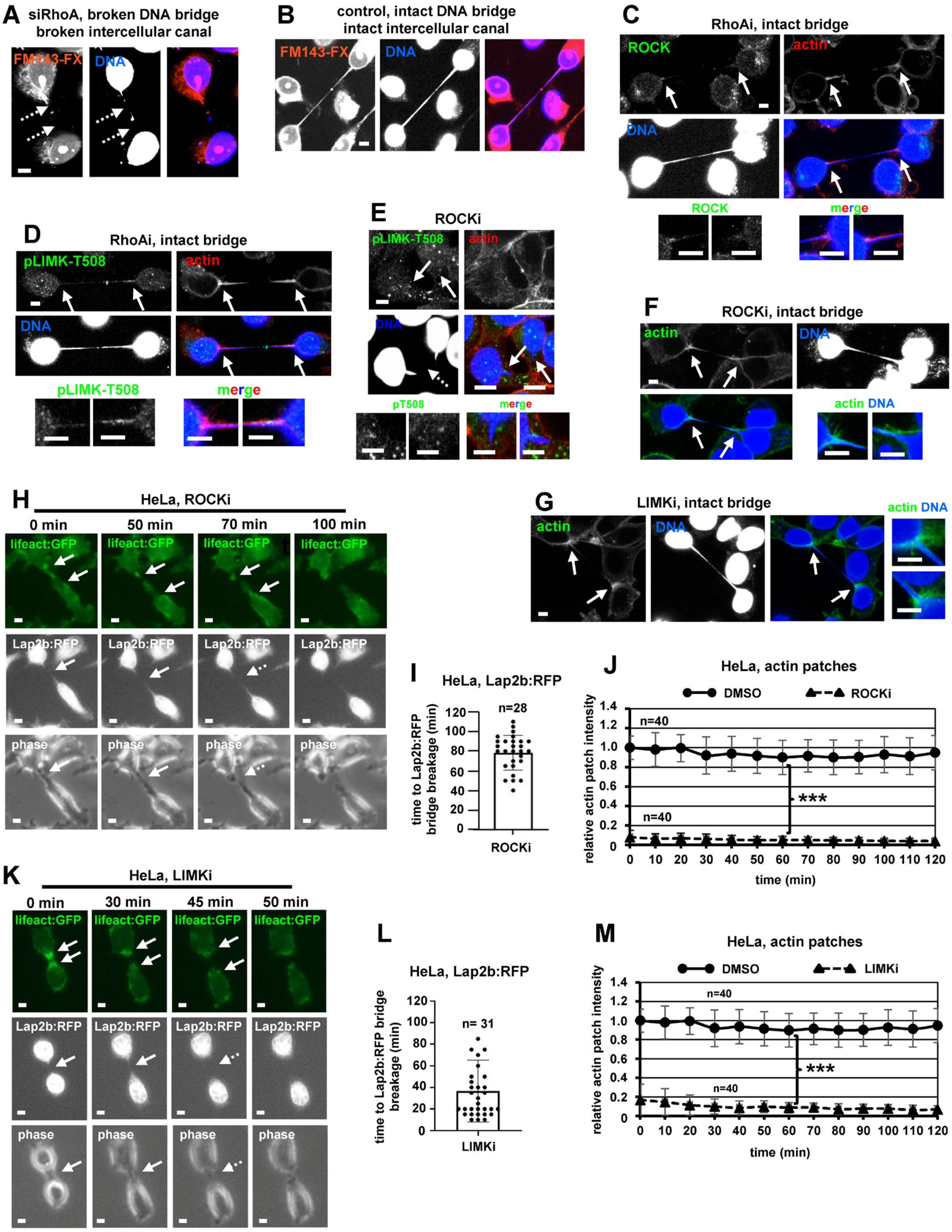

Appendix Figure S2. Inhibition of LIMK impairs actin patches and correlates with chromatin bridge breakage in cytokinesis. (A, B) Examples of BE cells with DNA bridges exhibiting broken or intact intercellular canals after labeling with the FM 1-43FX lipophilic dye. **(C-E)** Localization of ROCK and phosphorylated LIMK-T508 (pT508) after treatment of cells with 50 μM Y16 (RhoAi) or 10 μM Y27632 (ROCKi) for 1 h. **(F, G)** Actin patches and chromatin bridges in cells treated with ROCKi for 1 h or 10 μM TH-257 (LIMKi) for 5 h. **(H, K)** HeLa cells expressing Lap2b:RFP and Lifeact:GFP were analyzed by live-cell imaging in the presence of ROCKi or LIMKi. Time is from the detection of Lap2b:RFP bridges. Intact arrows indicate actin patches, intact DNA bridges or intercellular canals. Broken arrows indicate broken DNA bridges or intercellular canals. Insets show high magnifications of the canal bases. Bars, 5 μm. **(I, L)** Time to Lap2b:RFP bridge breakage. Mean ± SD from n cells. **(J)** Intensity of actin patches in cells treated with ROCKi or DMSO. Mean ± SD from n cells. ****P*= 2.04E-44 (DMSO vs ROCKi, 60 min) by Student's t test. **(M)** Intensity of actin patches in cells treated with LIMKi or DMSO. Mean ± SD from n cells. ****P*= 2.28E-42 (DMSO vs LIMKi, 60 min) by Student's t test.

Appendix Figure S3

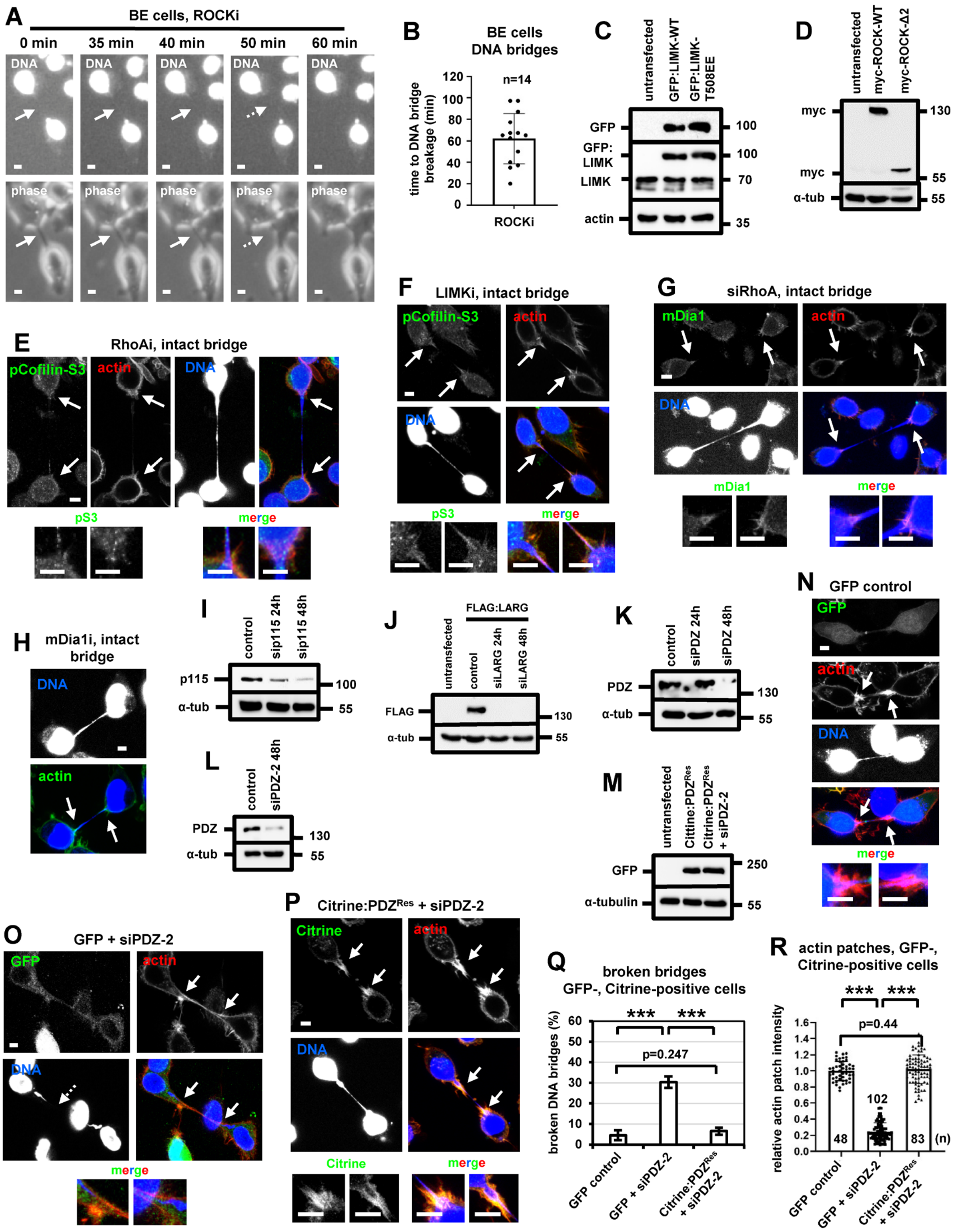

Appendix Figure S3. Expression of siRNA-resistant Citrine:PDZ rescues actin patches and prevents chromatin bridge breakage in cytokinesis. (A) BE cells labelled with Biotracker DNA dye were analyzed by live-cell imaging in the presence of 10 μM Y27632 (ROCKi). Time is from the detection of DNA bridges. **(B)** Time to DNA bridge breakage. Mean ± SD from n cells. **(C, D)** Western blot analysis of total GFP, LIMK, myc, actin or α-tubulin (α-tub). **(E, F)** Localization of phosphorylated Cofilin-S3 (pCofilin-S3) in cells with intact DNA bridges treated with 50 μM Y16 (RhoAi) for 1 h, or 10 μM TH-257 (LIMKi) for 5 h. **(G)** Localization of mDia1 in cells transfected with RhoA siRNA (siRhoA). **(H)** Actin patches in cells treated with 25 μM SMIFH2 (mDia1i) for 5 h. **(I-M)** Western blot analysis of total p115, FLAG, PDZ RhoGEF, GFP or α-tubulin. **(N-P)** Actin patches and DNA bridges in cells expressing GFP or siRNA-resistant Citrine:PDZ (Citrine:PDZ^{Res}) in the absence (control) or presence of PDZ siRNA-2 (siPDZ-2). Intact arrows indicate actin patches and intact Lap2b:RFP bridges or intercellular canals. Broken arrows indicate broken Lap2b:RFP bridges, DNA bridges or intercellular canals. Insets show high magnifications of the canal bases. Bars, 5 μm. **(Q)** Percentage of broken DNA bridges. Mean ± SD from three independent experiments (n= 125, 56, 60). ****P*= 1.86E-06 (GFP control vs GFP+siPDZ-2), 0.00022 (GFP+siPDZ-2 vs Citrine:PDZ^{Res}+siPDZ-2) by ANOVA and Student's t test. **(R)** Actin patches intensity. Mean ± SD from n cells from two independent experiments. Values in GFP control were set to 1. Numbers below/next to each bar indicate n. ****P*= 2.09E-78 (GFP control vs GFP+siPDZ-2), 3.54E-86 (GFP+siPDZ-2 vs Citrine:PDZ^{Res}+siPDZ-2) by ANOVA and Student's t test.

Appendix Figure S4

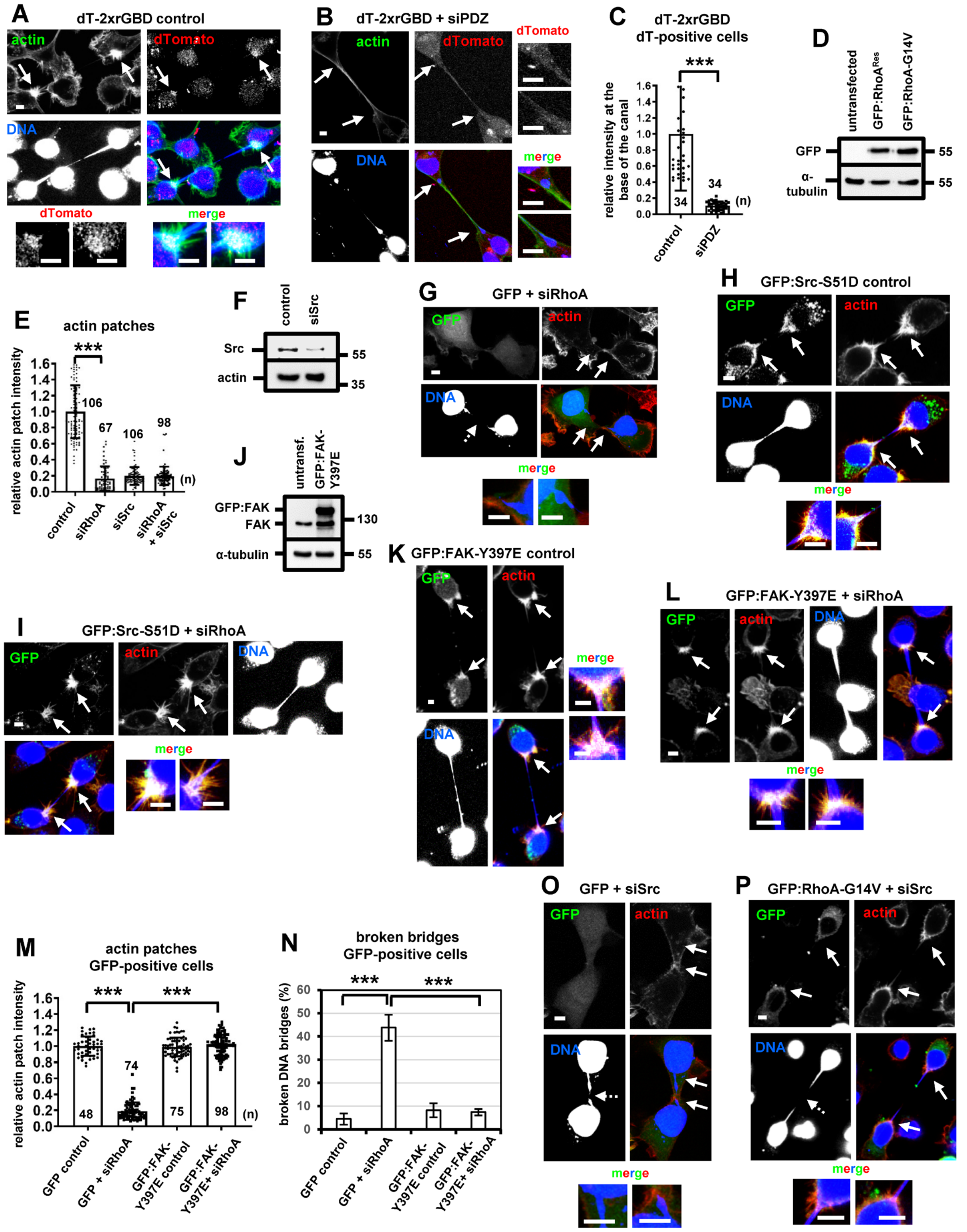

Appendix Figure S4. Expression of constitutively active GFP:FAK-Y397E rescues actin patches and prevents chromatin bridge breakage in RhoA-deficient cells. (A, B) Localization of dimericTomato-2xrGBD (dT-2xrGBD) Rho biosensor in BE cells in the absence (control) or presence of PDZ siRNA (siPDZ). **(C)** dT-2xrGBD fluorescence intensity. Mean ± SD from n cells from two independent experiments. ****P*= 3.60E-10 by Student's t test. **(D)** Western blot analysis of total GFP or α-tubulin. **(E)** Actin patches intensity. Mean ± SD from n cells from two independent experiments. Values in control were set to 1. ****P*= 5.51E-45 by ANOVA and Student's t test. **(F)** Western blot analysis of total Src or actin. **(G-I)** Actin patches and chromatin bridges in cells transfected with GFP or GFP:Src-S51D in the abscence (control) or presence of RhoA siRNA (siRhoA). **(J)** Western blot analysis of total FAK or α-tubulin. **(K, L)** Actin patches and chromatin bridges in cells transfected with GFP:FAK-Y397E. **(M)** Actin patches intensity in GFP-positive cells. Mean ± SD from n cells from two independent experiments. Values in GFP control were set to 1. Numbers below/next to each bar indicate n. ****P*= 1.84E-69 (GFP control vs GFP+siRhoA), 4.35E-94 (GFP+siRhoA vs GFP:FAK-Y397E+siRhoA) by ANOVA and Student's t test. **(N)** Percentage of broken DNA bridges. Mean ± SD from three independent experiments (n= 125, 89, 61, 78). ****P*= 1.32E-07 (GFP control vs GFP+siRhoA), 6.98E-05 (GFP+siRhoA vs GFP:FAK-Y397E+siRhoA) by ANOVA and Student's t test. **(O, P)** Actin patches and chromatin bridges in cells expressing GFP or GFP:RhoA-G14V in the presence of Src siRNA (siSrc). Intact arrows indicate actin patches or canal bases. Broken arrows indicate broken DNA bridges. Insets show high magnifications of the canal bases. Bars, 5 μm.

Appendix Figure S5

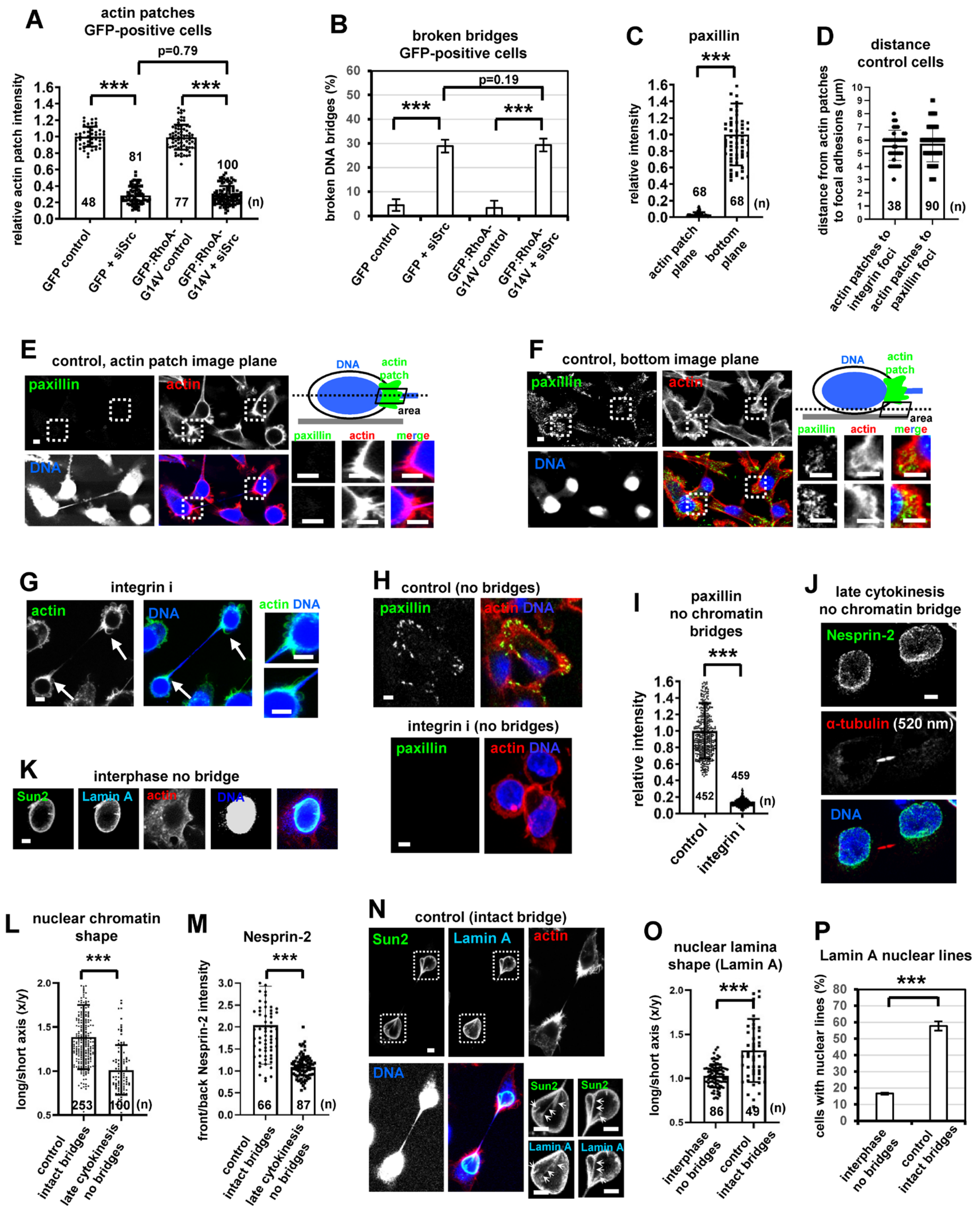

Appendix Figure S5. Expression of constitutively active GFP:RhoA-G14V does not rescue actin patches in Src-deficient cells. (A) Actin patches intensity in GFP-positive cells. BE cells were expressing GFP:RhoA-G14V or GFP-only in the absence (control) or presence of Src siRNA (siSrc). Mean ± SD from n cells from two independent experiments. Values in GFP control were set to 1. ***P= 1.39E-66 (GFP control vs GFP+siSrc), 8.06E-82 (GFP:RhoA-G14V+siSrc) by ANOVA and Student's t test. (**B**) Percentage of broken DNA bridges. Mean ± SD from three independent experiments (n= 125, 61, 83, 75). ***P= 1.55E-05 (GFP control vs GFP+siSrc), 0.00024 (GFP:RhoA-G14V+siSrc) by ANOVA and Student's t test. **(C)** Paxillin intensity at the actin patches image plane or at the bottom plane in control cells with chromatin bridges. Mean ± SD from n cells from two independent experiments. Values in bottom plane were set to 1. ***P= 8.22E-45 by Student's t test. **(D)** Distance from actin patches to integrin or paxillin foci. Mean ± SD from n cells from two independent experiments. **(E, F)** Localization of paxillin at the actin patch image plane or the cell bottom plane of the same cells, grown on fibronectin-coated slides. Dotted lines indicate the depicted image planes; rectangles indicate areas magnified in insets. **(G, H)** Actin patches and paxillin localization in the absence of drugs (control) or in cells treated with 1 μM SB273005 (integrin i) for 15 min. **(I)** Paxilin intensity in interphase cells without DNA bridges. Mean ± SD from n cells from two independent experiments. Values in control were set to one. ***P= 3.06E-292 by Student's t test. **(J)** Nesprin-2 localization in cells in late cytokinesis connected by a late midbody. Tubulin values indicate midbody thickness. **(K)** Sun2 and Lamin A localization in interphase cells. **(L)** Nuclear chromatin shape in control cells with intact DNA bridges and in cells in late cytokinesis without DNA bridges. Mean ± SD from n cells from two independent experiments. ***P= 2.58E-18 by Student's t test. **(M)** Front/back Nesprin-2 intensity. Mean ± SD from n cells from two independent experiments. ***P= 5.57E-17 by Student's t test. **(N)** Sun2 and Lamin A localization in control cells with intact DNA bridges. Arrowheads show Lamin A nuclear lines. Insets show high magnifications of boxed areas or canal bases. **(O)** Nuclear lamina shape after Lamin A staining. Mean ± SD from n cells from two independent experiments. Numbers below/next to each bar indicate n. ***P= 3.88E-10 by Student's t test. **(P)** Percentage of cells exhibiting Lamin A nuclear lines. Mean ± SD from three independent experiments (n= 196, 50). ***P= 1.61E-05 by Student's t test. Bars, 5 μm**.**

Appendix Figure S6

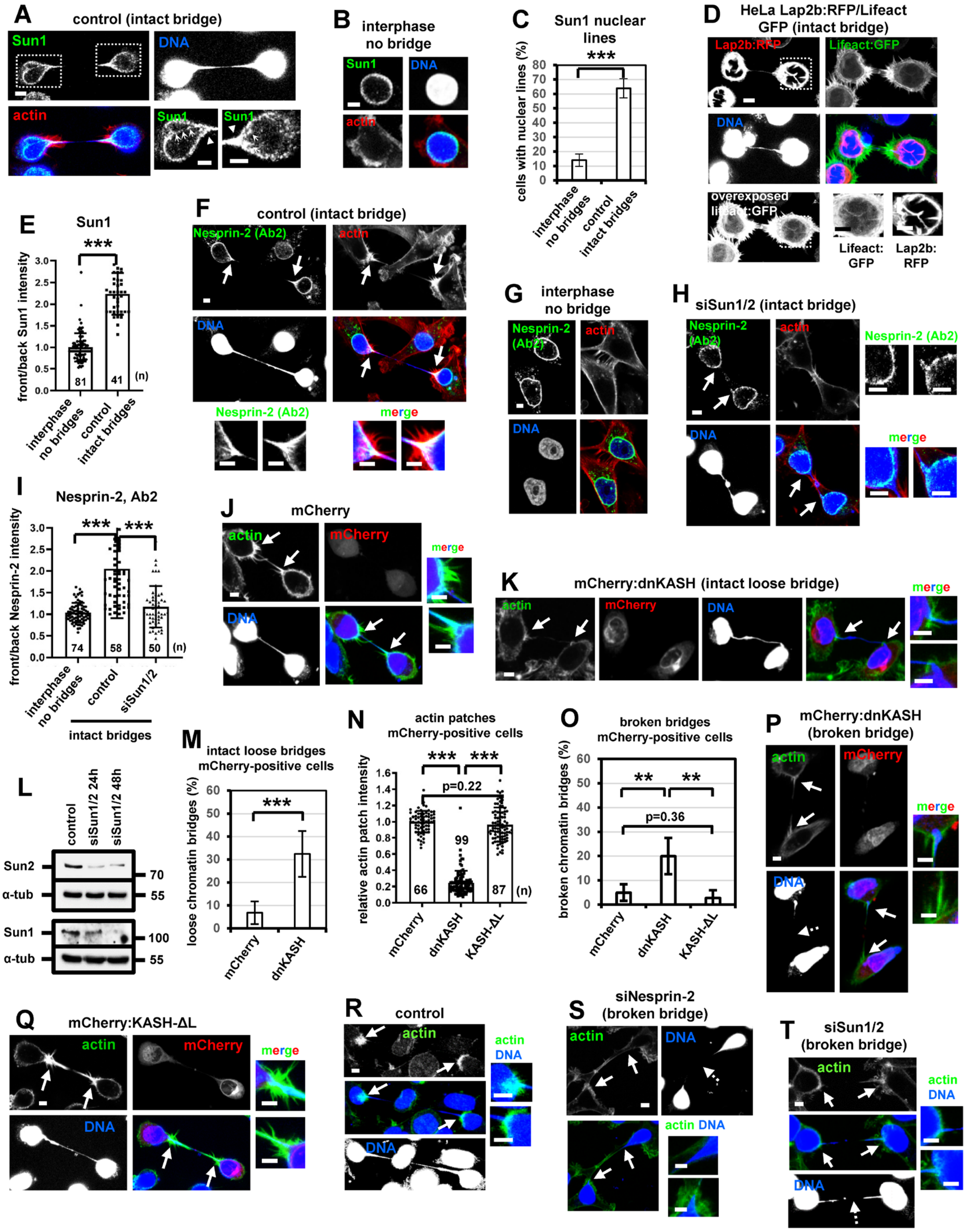

Appendix Figure S6. Expression of dominant-negative (dn) KASH domain impairs actin patches and correlates with increased frequency of broken chromatin bridges in cytokinesis. (A, B) Sun1 localization in BE cells. Arrowheads show Sun1 nuclear lines and accumulations. **(C)** Percentage of cells exhibiting Sun1 nuclear lines. Mean ± SD from three independent experiments (n= 310, 50). ****P*= 0.00041 by Student's t test. **(D)** HeLa cells expressing Lap2b:RFP and Lifeact:GFP and exhibiting an intact Lap2b:RFP bridge were fixed and analyzed by confocal microscopy. To detect actin nuclear lines, the Lifeact:GFP image was overexposed by increasing the brightness and contrast by 40%. Image is representative of 10 cells from two independent experiments. **(E)** Front/back Sun1 intensity in BE cells. Mean ± SD from n cells from two independent experiments. ****P*= 6.05E-33 by Student's t test. **(F-H)** Nesprin-2 localization after staining with an N-terminal Nesprin-2 antibody (Ab2), in the absence (control) or presence of Sun1/2 siRNA (siSun1/2). **(I)** Front/back Nesprin-2 intensity. Mean ± SD from n cells from two independent experiments. ****P*= 1.09E-11 (interphase no bridges vs control intact bridges), 1.68E-06 (control intact bridges vs siSun1/2 intact bridges) by ANOVA and Student's t test. **(J, K)** Actin patches and chromatin bridges in cells expressing mCherry or dnKASH. **(L)** Western blot analysis of total Sun1, Sun2 or α-tubulin (α-tub). **(M)** Percentage of intact loose DNA bridges in mCherry-positive cells. Mean ± SD from three independent experiments (n= 107, 100). ****P*= 0.00034 by Student's t test. **(N)** Actin patches intensity. Mean ± SD from n cells from two independent experiments. Values in mCherry were set to 1. Numbers below/next to each bar indicate n. ****P*= 8.52E-75 (mCherry vs dnKASH), 4.47E-66 (dnKASH vs KASH-ΔL) by ANOVA and Student's t test. **(O)** Percentage of broken DNA bridges. Mean ± SD from three independent experiments (n= 57, 100, 52). ***P*= 0.0078 (mCherry vs dnKASH), 0.0036 (dnKASH vs KASH-ΔL) by ANOVA and Student's t test. **(P, Q)** Actin patches and chromatin bridges in cells expressing dnKASH or KASH-ΔL. **(R-T)** Actin patches and chromatin bridges in the absence (control) or presence of Nesprin-2 siRNA (siNesprin-2) or siSun1/2. Intact arrows indicate actin patches or canal bases. Broken arrows indicate broken DNA bridges. Insets show high magnifications of boxed areas or canal bases. Bars, 5 μm.

Appendix Figure S7

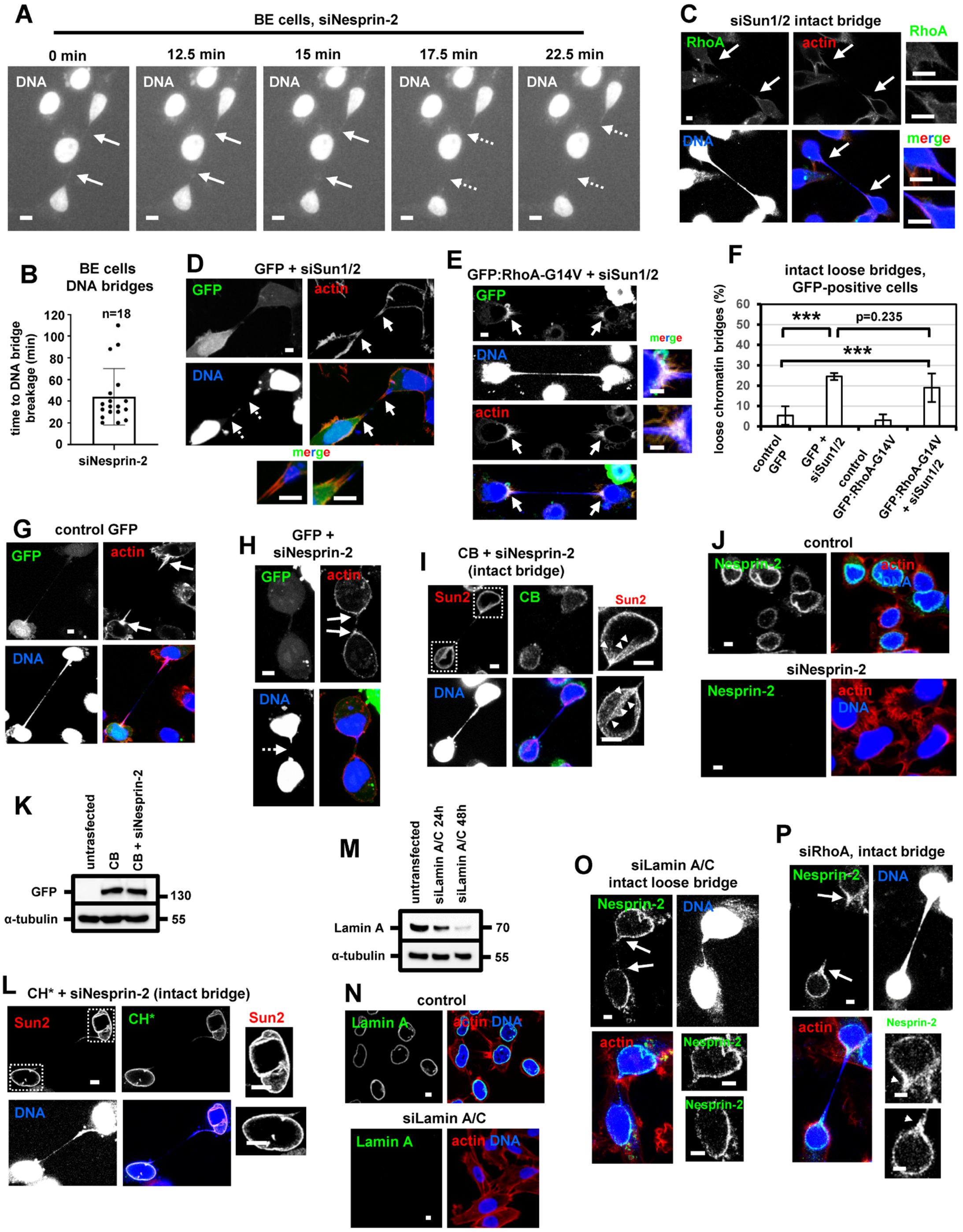

Appendix Figure S7. Depletion of Nesprin-2 correlates with chromatin bridge breakage in living cells. (A) BE cells transfected with Nesprin-2 siRNA (siNesprin-2) were labelled with Biotracker DNA dye and analyzed by live-cell imaging. Time is from the detection of DNA bridges. Intact arrows indicate intact DNA bridges and broken arrows indicate broken DNA bridges. **(B)** Time to DNA bridge breakage. Mean ± SD from n cells. **(C)** RhoA localization in cells expressing Sun1/2 siRNA (siSun1/2). **(D, E)** Actin patches and DNA bridges in cells expressing GFP or GFP:RhoA-G14V, in the presence of siSun1/2. **(F)** Percentage of intact loose DNA bridges in GFP-positive cells. Mean ± SD from three independent experiments (n= 143, 57, 83, 71). ****P*= 3.32E-05 (control GFP vs GFP+siSun1/2), 0.00075 (control GFP vs GFP:RhoA-G14V+siSun1/2) by ANOVA and Student's t test. **(G, H)** Actin patches and DNA bridges in cells expressing GFP in the absence (control) or presence of siNesprin-2. **(I)** Sun2 localization in cells expressing mini-Nesprin-2 CB. **(J)** Nesprin-2 localization. **(K)** Western blot analysis of total GFP and α-tubulin. **(L)** Sun2 localization in cells expressing mini-Nesprin-2 CH*. **(M)** Western blot analysis of total Lamin A and α-tubulin. **(N)** Lamin A localization in the absence (control) or presence of Lamin A/C siRNA (siLamin A/C) at 48 h post-transfection. **(O, P)** Nesprin-2 localization in cells transfected with siLamin A/C or RhoA siRNA (siRhoA). Intact arrows indicate the bases of the intercellular canals. Arrowheads show Sun2 nuclear lines or accumulations of Nesprin-2. Insets show high magnifications of boxed areas or canal bases. Bars, 5 μm.

Appendix Figure S8

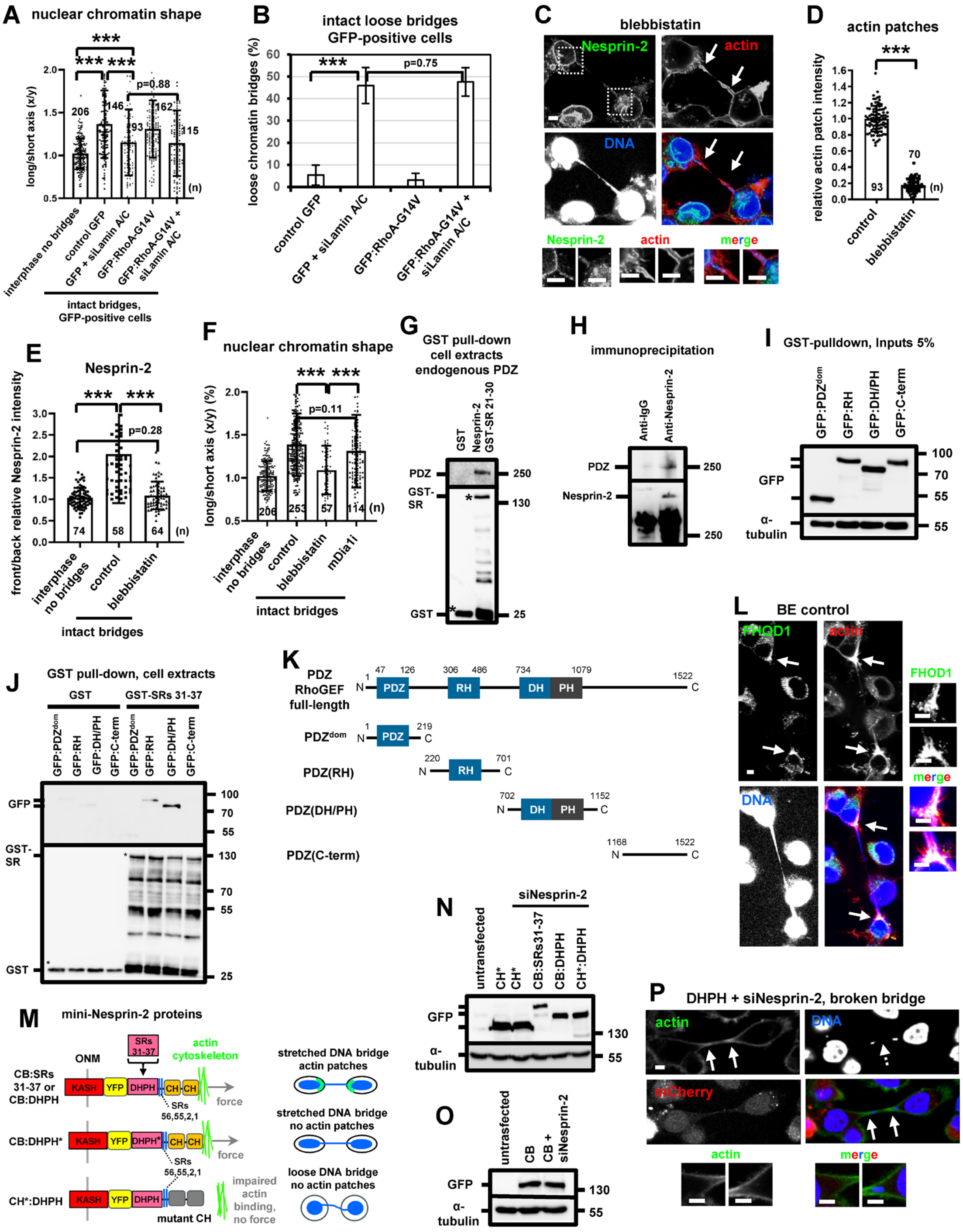

Appendix Figure S8. Nesprin-2 Spectrin Repeats 31-37 associate with the RH and DH/PH domains of PDZ RhoGEF. (A) Nuclear chromatin shape in interphase BE cells without DNA bridges, or in cells with intact DNA bridges expressing GFP or GFP:RhoA-G14V in the absence (control) or presence of LaminA/C siRNA (siLaminA/C). Mean ± SD from from n cells from two independent experiments. ****P*= 1.01E-24 (interphase no bridges vs control GFP intact bridges), 6.45E-05 (interphase no bridges vs GFP+siLaminA/C intact bridges), 5.53E-05 (control GFP intact bridges vs GFP+siLaminA/C intact bridges) by ANOVA and Student's t test. **(B)** Percentage of cells exhibiting intact loose chromatin bridges. Mean ± SD from three independent experiments (n= 143, 58, 83, 63). ****P*= 5.75E-07 by ANOVA and Student's t test. **(C)** Nesprin-2 localization in cells treated with 50 µM blebbistatin for 30 min. **(D)** Actin patches intensity. Mean ± SD from n cells from two independent experiments. Values in control were set to 1. ****P*= 4.09E-85 by Student's t test. **(E)** Front/back Nesprin-2 intensity. Mean ± SD from n cells from two independent experiments. ****P*= 1.09E-11 (interphase no bridges vs control intact bridges), 2.22E-09 (control intact bridges vs blebbistatin intact bridges) by ANOVA and Student's t test. **(F)** Nuclear chromatin shape in cells treated as in E, or with 25 µM SMIFH2 (mDia1i) for 5 h. Mean ± SD from from n cells from two independent experiments. Numbers below/next to each bar indicate n. ****P*= 2.17E-08 (control intact bridges vs blebbistatin intact bridges), 0.00030 (blebbistatin intact bridges vs mDia1i intact bridges) by ANOVA and Student's t test. **(G)** BE cell lysates were incubated with GST-SRs 21-30 or GST-only. Associated proteins were detected by Western blotting. **(H)** Immunoprecipitation of Nesprin-2 and PDZ RhoGEF in BE cell lysates using the N-terminal Nesprin-2 antibody (Ab2). **(I)** Western blot analysis of total GFP and α-tubulin in cell lysates for GST pull-downs. **(J)** Cell lysates from cells expressing truncated PDZ RhoGEFs fused to GFP were incubated with Nesprin-2 GST-SRs 31-37 or with GST-only. Associated proteins were detected by Western blotting. **(K)** Cartoons of human PDZ RhoGEF protein constructs. **(L)** FHOD1 localization. **(M)** Cartoons of mini-Nesprin-2 proteins. Nesprin-2 SRs 31-37 or the DHPH domain of PDZ RhoGEF were inserted between the YFP and the neighboring spectrin repeat of CB or CH*. SRs, spectrin repeats; ONM, outer nuclear membrane; CH, calponin homology; DHPH, Dbl homology/pleckstrin homology; DHPH*, mutant DHPH-E15A. **(N, O)** Western blot analysis of total GFP and α-tubulin. **(P)** Actin patches in cells expressing DHPH fused to mCherry in the presence of Nesprin-2 siRNA

(siNesprin-2). Intact arrows indicate the bases of the intercellular canals. Broken arrows indicate broken DNA bridges. Insets show high magnifications of boxed areas or canal bases. Bars, 5 μm.

Appendix Figure S9

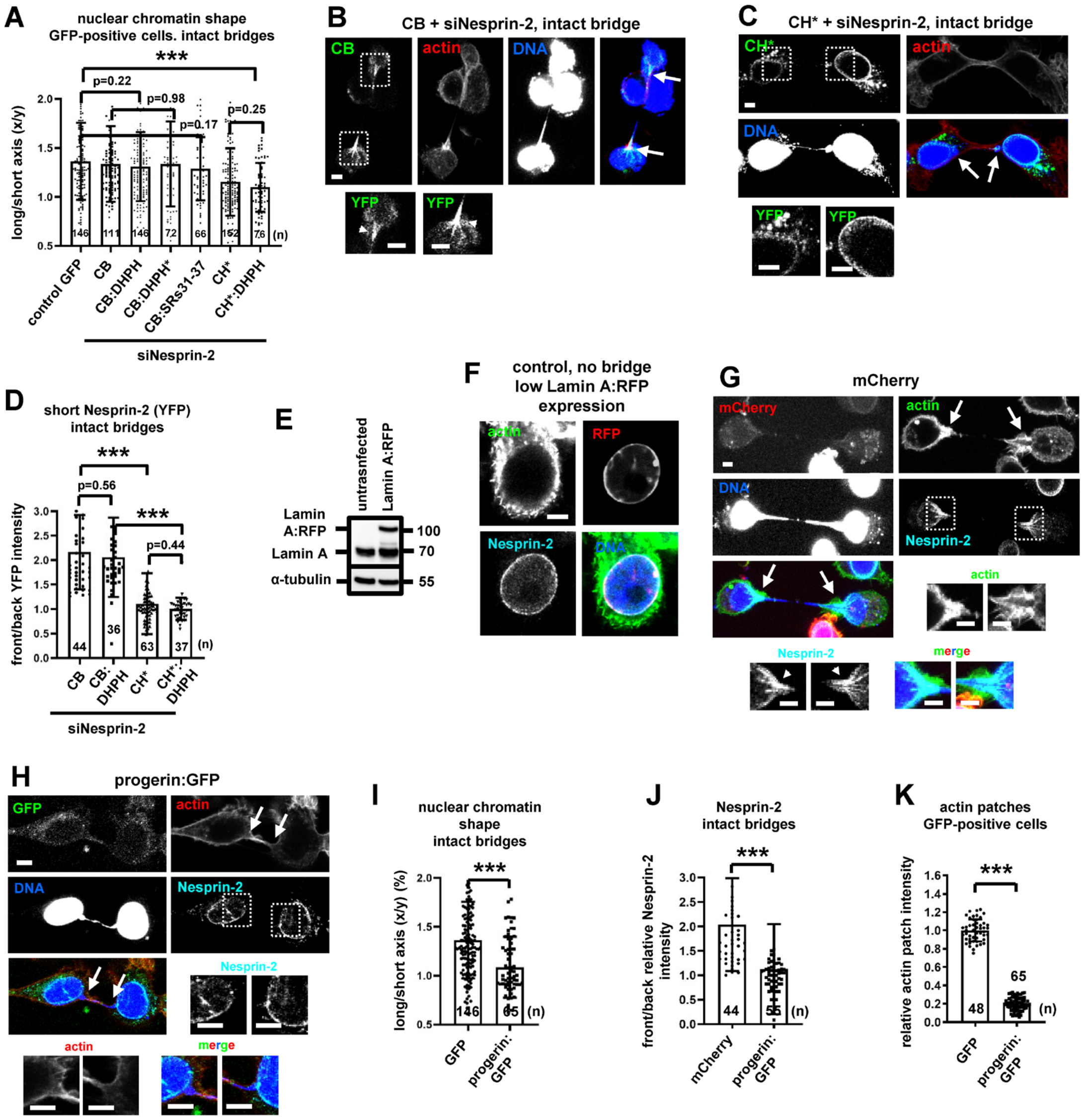

Appendix Figure S9. Expression of mini-Nesprin-2 CH* or CH*:DHPH proteins diminishes nuclear chromatin shape-deformation in cytokinesis with intact chromatin bridges. (A) Nuclear chromatin shape in BE cells expressing GFP or mini-Nesprin-2 proteins in the absence (control) or presence of Nesprin-2 siRNA (siNesprin-2). Mean ± SD from n cells from two independent experiments. ****P*= 9.08E-07 by ANOVA and Student's t test. **(B, C)** Mini-Nesprin-2 CB or CH* localization. **(D)** Front/back mini-Nesprin-2 intensity. Mean ± SD from n cells from two independent experiments. ****P*= 3.71E-12 (CB siNesprin-2 vs CH* siNesprin-2), 9.38E-11 (CB:DHPH siNesprin-2 vs CH*:DHPH siNesprin-2) by ANOVA and Student's t test. **(E)** Western blot analysis of total Lamin A or α-tubulin. **(F)** Localization of Lamin A:RFP. **(G, H)** Actin patches and Nesprin-2 localisation in cells expressing mCherry-only or progerin:GFP. Intact arrows indicate the bases of the intercellular canals. Insets show high magnifications of the canal bases. Arrowheads show accumulation of the endogenous Nesprin-2 or mini-Nesprin-2 proteins. **(I)** Nuclear chromatin shape. Mean ± SD from n cells from two independent experiments. ****P*= 1.09E-06 by Student's t test. **(J)** Front/back mini-Nesprin-2 intensity. Mean ± SD from n cells from two independent experiments. ****P*= 5.53E-06 by Student's t test. **(K)** Actin patches intensity. Mean ± SD from n cells from two independent experiments. Values in GFP were set to 1. Numbers below/next to each bar indicate n. ****P*= 6.30E-72 by Student's t test. Bars, 5 μm.
