# Supplementary material for: Tension-sensitive LINC-RhoA signaling prevents chromatin bridge breakage in cytokinesis
Source: EMBO J. 2025 Sep 9;44(20):5834–59. doi: 10.1038/s44318-025-00565-3 (PMC12528419; doi:10.1038/s44318-025-00565-3)
Supplement: Supplementary file 5 — Movie EV3 [file 44318_2025_565_MOESM5_ESM.zip › Movie EV3 legend.docx]

**Movie EV3. Control cells exhibit stable intercellular canals in cytokinesis.** HeLa cells stably expressing Lifeact:GFP and Lap2b:RFP were were treated with 50 μΜ DMSO and analyzed by phase-contrast time-lapse microscopy. Frames were taken every 10 min for 130 min. Time counters show minutes: seconds. Display rate: one frame per second. Related image stills are shown in Figure 1G.
