# Supplementary material for: Tension-sensitive LINC-RhoA signaling prevents chromatin bridge breakage in cytokinesis
Source: EMBO J. 2025 Sep 9;44(20):5834–59. doi: 10.1038/s44318-025-00565-3 (PMC12528419; doi:10.1038/s44318-025-00565-3)
Supplement: Supplementary file 6 — Movie EV4 [file 44318_2025_565_MOESM6_ESM.zip › Movie EV4 legend.docx]

**Movie EV4. Impaired actin patches after RhoA inhibition.** HeLa cells stably expressing Lifeact:GFP (green) and Lap2b:RFP were treated with 50 μΜ Y16 (RhoAi) and analyzed by time-lapse fluorescence microscopy. Frames were taken every 10 min for 150 min. Time counters show minutes: seconds. Display rate: one frame per second. Related image stills are shown in Figure 1H.
