# Supplementary material for: Tension-sensitive LINC-RhoA signaling prevents chromatin bridge breakage in cytokinesis
Source: EMBO J. 2025 Sep 9;44(20):5834–59. doi: 10.1038/s44318-025-00565-3 (PMC12528419; doi:10.1038/s44318-025-00565-3)
Supplement: Supplementary file 13 — Movie EV11 [file 44318_2025_565_MOESM13_ESM.zip › Movie EV11 legend.docx]

**Movie EV11. Impaired actin patches after ROCK inhibition.** HeLa cells stably expressing Lifeact:GFP (green) and Lap2b:RFP were treated with 10 μΜ Y27632 (ROCKi) and analyzed by time-lapse fluorescence microscopy. Frames were taken every 5 min for 105 min. Time counters show minutes: seconds. Display rate: one frame per second. Related image stills are shown in Appendix Figure S2H.
