# Supplementary material for: Tension-sensitive LINC-RhoA signaling prevents chromatin bridge breakage in cytokinesis
Source: EMBO J. 2025 Sep 9;44(20):5834–59. doi: 10.1038/s44318-025-00565-3 (PMC12528419; doi:10.1038/s44318-025-00565-3)
Supplement: Supplementary file 21 — Movie EV19 [file 44318_2025_565_MOESM21_ESM.zip › Movie EV19 legend.docx]

**Movie EV19. Actin patches are all around the base of the chromatin bridge**. BE cells with a chromatin bridge and actin patches were analyzed by confocal microscopy and a 3D reconstruction of image stacks was generated. Green: actin, blue: DNA. Display rate: two frames per second.
