# Supplementary material for: Tension-sensitive LINC-RhoA signaling prevents chromatin bridge breakage in cytokinesis
Source: EMBO J. 2025 Sep 9;44(20):5834–59. doi: 10.1038/s44318-025-00565-3 (PMC12528419; doi:10.1038/s44318-025-00565-3)
Supplement: Supplementary file 22 — Movie EV20 [file 44318_2025_565_MOESM22_ESM.zip › Movie EV20 legend.docx]

**Movie EV20. Breakage of DNA bridges after Nesprin-2 depletion.** BE cells transfected with Nesprin-2 siRNA and labelled with Biotracker DNA dye (white) were analyzed by time-lapse fluorescence microscopy. Frames were taken every 2.5 min for 40 min. Time counters show minutes: seconds. Display rate: one frame per second. Related image stills are shown in Appendix Figure S7A.
