# Supplementary figures and images for: Tension-sensitive LINC-RhoA signaling prevents chromatin bridge breakage in cytokinesis (part 1 of 2)
Source: EMBO J. 2025 Sep 9;44(20):5834–59. doi: 10.1038/s44318-025-00565-3 (PMC12528419; doi:10.1038/s44318-025-00565-3)

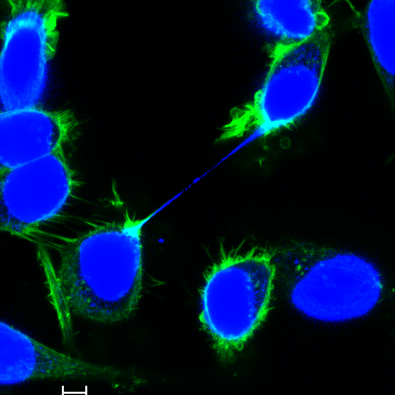

Supplement: Supplementary file 23 — Source data Fig. 1 [file 44318_2025_565_MOESM23_ESM.zip › Figure 1/1A/1A_microscopy.tif]

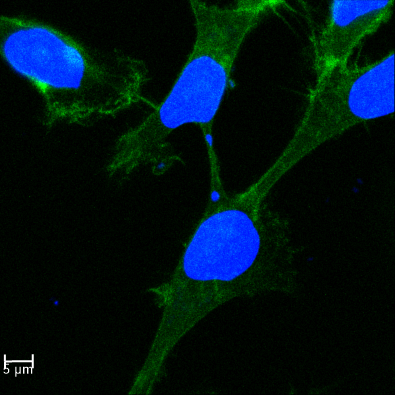

Supplement: Supplementary file 23 — Source data Fig. 1 [file 44318_2025_565_MOESM23_ESM.zip › Figure 1/1B/1B_microscopy.tif]

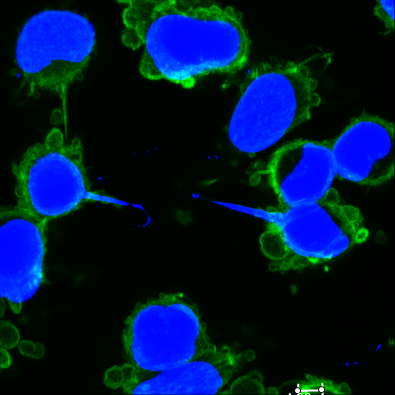

Supplement: Supplementary file 23 — Source data Fig. 1 [file 44318_2025_565_MOESM23_ESM.zip › Figure 1/1C/1C_microscopy.tif]

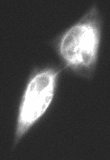

Supplement: Supplementary file 23 — Source data Fig. 1 [file 44318_2025_565_MOESM23_ESM.zip › Figure 1/1G/Lap2bRFP/0 min_microscopy.tif]

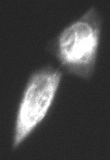

Supplement: Supplementary file 23 — Source data Fig. 1 [file 44318_2025_565_MOESM23_ESM.zip › Figure 1/1G/Lap2bRFP/100 min_microscopy.tif]

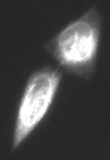

Supplement: Supplementary file 23 — Source data Fig. 1 [file 44318_2025_565_MOESM23_ESM.zip › Figure 1/1G/Lap2bRFP/120 min microscopy.tif]

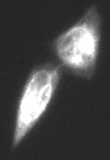

Supplement: Supplementary file 23 — Source data Fig. 1 [file 44318_2025_565_MOESM23_ESM.zip › Figure 1/1G/Lap2bRFP/30 min_microscopy.tif]

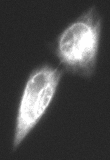

Supplement: Supplementary file 23 — Source data Fig. 1 [file 44318_2025_565_MOESM23_ESM.zip › Figure 1/1G/Lap2bRFP/70 min_microscopy.tif]

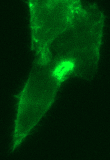

Supplement: Supplementary file 23 — Source data Fig. 1 [file 44318_2025_565_MOESM23_ESM.zip › Figure 1/1G/lifeactGFP/0 min_microscopy.tif]

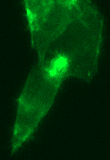

Supplement: Supplementary file 23 — Source data Fig. 1 [file 44318_2025_565_MOESM23_ESM.zip › Figure 1/1G/lifeactGFP/100 min microscopy.tif]

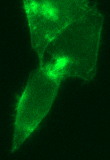

Supplement: Supplementary file 23 — Source data Fig. 1 [file 44318_2025_565_MOESM23_ESM.zip › Figure 1/1G/lifeactGFP/120 min_microscopy.tif]

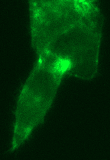

Supplement: Supplementary file 23 — Source data Fig. 1 [file 44318_2025_565_MOESM23_ESM.zip › Figure 1/1G/lifeactGFP/30 min-microscopy.tif]

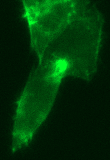

Supplement: Supplementary file 23 — Source data Fig. 1 [file 44318_2025_565_MOESM23_ESM.zip › Figure 1/1G/lifeactGFP/70 min_microscopy.tif]

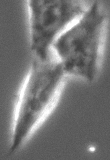

Supplement: Supplementary file 23 — Source data Fig. 1 [file 44318_2025_565_MOESM23_ESM.zip › Figure 1/1G/phase/0 min_microscopy.tif]

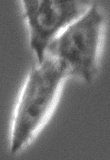

Supplement: Supplementary file 23 — Source data Fig. 1 [file 44318_2025_565_MOESM23_ESM.zip › Figure 1/1G/phase/100 min_microscopy.tif]

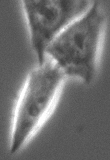

Supplement: Supplementary file 23 — Source data Fig. 1 [file 44318_2025_565_MOESM23_ESM.zip › Figure 1/1G/phase/120 min_microscopy.tif]

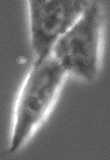

Supplement: Supplementary file 23 — Source data Fig. 1 [file 44318_2025_565_MOESM23_ESM.zip › Figure 1/1G/phase/30 min_microscopy.tif]

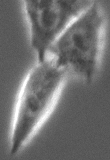

Supplement: Supplementary file 23 — Source data Fig. 1 [file 44318_2025_565_MOESM23_ESM.zip › Figure 1/1G/phase/70 min_microscopy.tif]

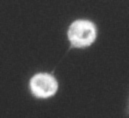

Supplement: Supplementary file 23 — Source data Fig. 1 [file 44318_2025_565_MOESM23_ESM.zip › Figure 1/1H/Lap2bRFP/0 min_microscopy.tif]

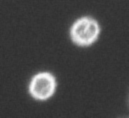

Supplement: Supplementary file 23 — Source data Fig. 1 [file 44318_2025_565_MOESM23_ESM.zip › Figure 1/1H/Lap2bRFP/100 min_microscopy.tif]

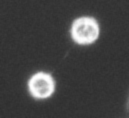

Supplement: Supplementary file 23 — Source data Fig. 1 [file 44318_2025_565_MOESM23_ESM.zip › Figure 1/1H/Lap2bRFP/30 min_microscopy.tif]

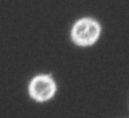

Supplement: Supplementary file 23 — Source data Fig. 1 [file 44318_2025_565_MOESM23_ESM.zip › Figure 1/1H/Lap2bRFP/70 min_microscopy.tif]

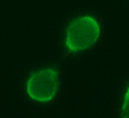

Supplement: Supplementary file 23 — Source data Fig. 1 [file 44318_2025_565_MOESM23_ESM.zip › Figure 1/1H/LifeactGFP/0 min_microscopy.tif]

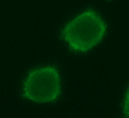

Supplement: Supplementary file 23 — Source data Fig. 1 [file 44318_2025_565_MOESM23_ESM.zip › Figure 1/1H/LifeactGFP/100 min_microscopy.tif]

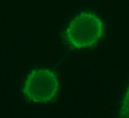

Supplement: Supplementary file 23 — Source data Fig. 1 [file 44318_2025_565_MOESM23_ESM.zip › Figure 1/1H/LifeactGFP/30 min_microscopy.tif]

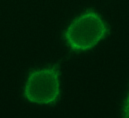

Supplement: Supplementary file 23 — Source data Fig. 1 [file 44318_2025_565_MOESM23_ESM.zip › Figure 1/1H/LifeactGFP/70 min_microscopy.tif]

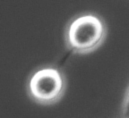

Supplement: Supplementary file 23 — Source data Fig. 1 [file 44318_2025_565_MOESM23_ESM.zip › Figure 1/1H/phase/0 min_microscopy.tif]

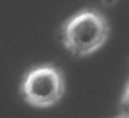

Supplement: Supplementary file 23 — Source data Fig. 1 [file 44318_2025_565_MOESM23_ESM.zip › Figure 1/1H/phase/100 min_microscopy.tif]

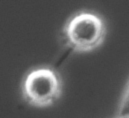

Supplement: Supplementary file 23 — Source data Fig. 1 [file 44318_2025_565_MOESM23_ESM.zip › Figure 1/1H/phase/30 min microscopy.tif]

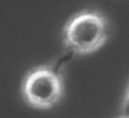

Supplement: Supplementary file 23 — Source data Fig. 1 [file 44318_2025_565_MOESM23_ESM.zip › Figure 1/1H/phase/70 min_microscopy.tif]

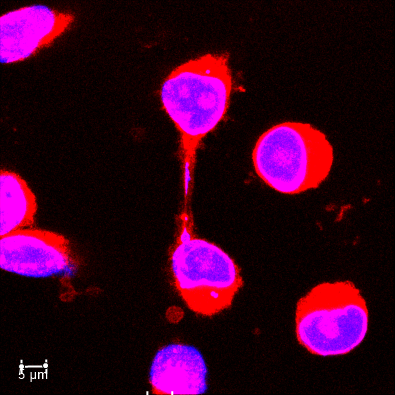

Supplement: Supplementary file 23 — Source data Fig. 1 [file 44318_2025_565_MOESM23_ESM.zip › Figure 1/1K/1K_microscopy.tif]

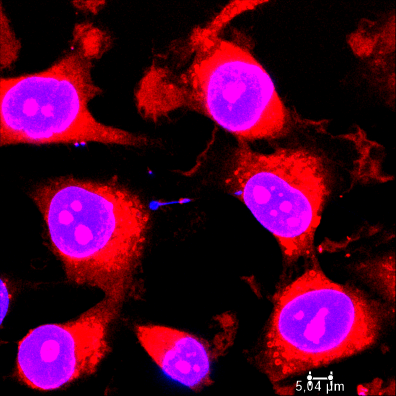

Supplement: Supplementary file 23 — Source data Fig. 1 [file 44318_2025_565_MOESM23_ESM.zip › Figure 1/1L/1L_microscopy.tif]

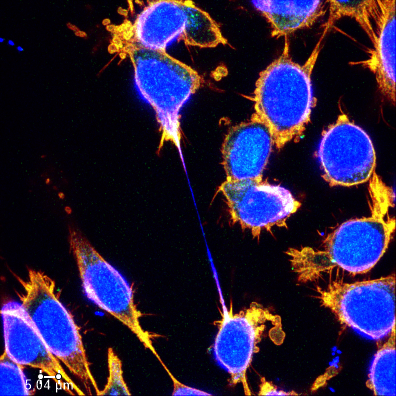

Supplement: Supplementary file 23 — Source data Fig. 1 [file 44318_2025_565_MOESM23_ESM.zip › Figure 1/1N/1N_microscopy.tif]

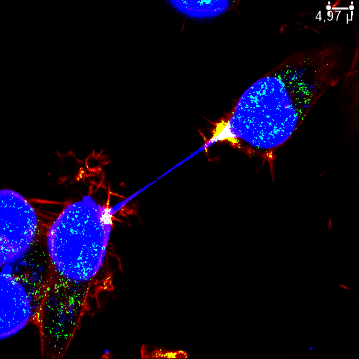

Supplement: Supplementary file 24 — Source data Fig. 2 [file 44318_2025_565_MOESM24_ESM.zip › Figure 2/2A/2A_microscopy.tif]

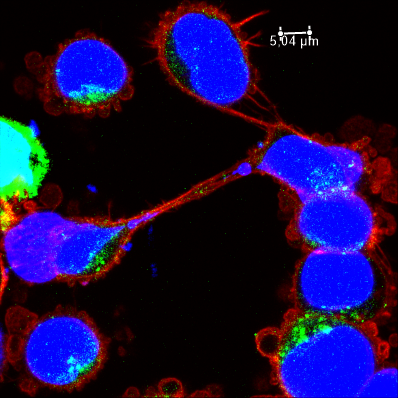

Supplement: Supplementary file 24 — Source data Fig. 2 [file 44318_2025_565_MOESM24_ESM.zip › Figure 2/2B/2B_microscopy.tif]

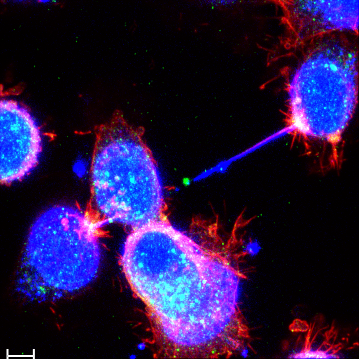

Supplement: Supplementary file 24 — Source data Fig. 2 [file 44318_2025_565_MOESM24_ESM.zip › Figure 2/2D/2D_microscopy.tif]

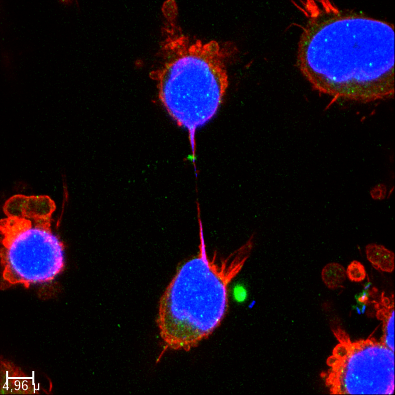

Supplement: Supplementary file 24 — Source data Fig. 2 [file 44318_2025_565_MOESM24_ESM.zip › Figure 2/2E/2E_microscopy.tif]

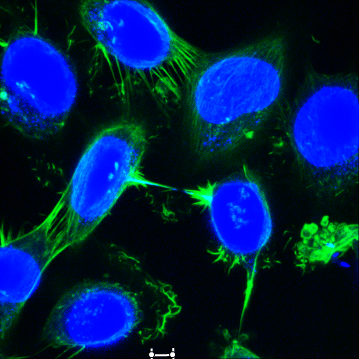

Supplement: Supplementary file 24 — Source data Fig. 2 [file 44318_2025_565_MOESM24_ESM.zip › Figure 2/2G/2G_microscopy.tif]

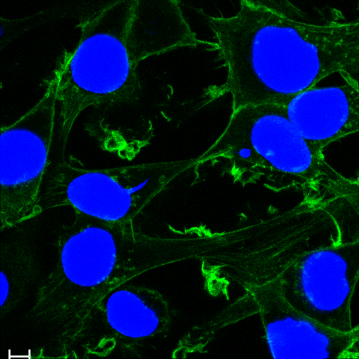

Supplement: Supplementary file 24 — Source data Fig. 2 [file 44318_2025_565_MOESM24_ESM.zip › Figure 2/2H/2H_microscopy.tif]

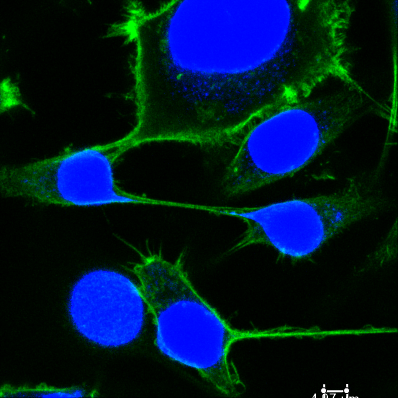

Supplement: Supplementary file 24 — Source data Fig. 2 [file 44318_2025_565_MOESM24_ESM.zip › Figure 2/2I/2I_microscopy.tif]

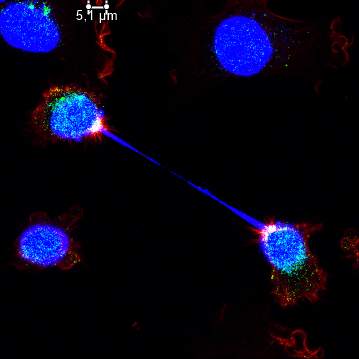

Supplement: Supplementary file 24 — Source data Fig. 2 [file 44318_2025_565_MOESM24_ESM.zip › Figure 2/2L/2L_microscopy.tif]

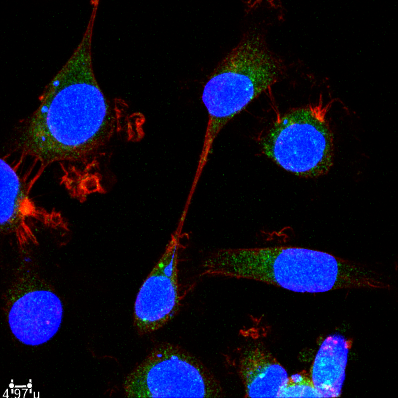

Supplement: Supplementary file 24 — Source data Fig. 2 [file 44318_2025_565_MOESM24_ESM.zip › Figure 2/2M/2M_microscopy.tif]

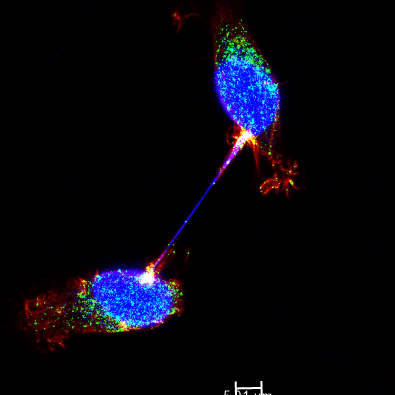

Supplement: Supplementary file 24 — Source data Fig. 2 [file 44318_2025_565_MOESM24_ESM.zip › Figure 2/2N/2N_microscopy.tif]

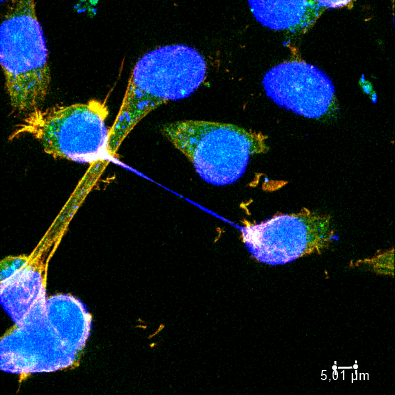

Supplement: Supplementary file 24 — Source data Fig. 2 [file 44318_2025_565_MOESM24_ESM.zip › Figure 2/2O/2O_microscopy.tif]

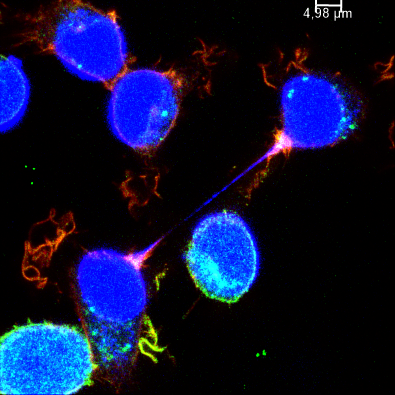

Supplement: Supplementary file 25 — Source data Fig. 3 [file 44318_2025_565_MOESM25_ESM.zip › Figure 3/3A/3A_microscopy.tif]

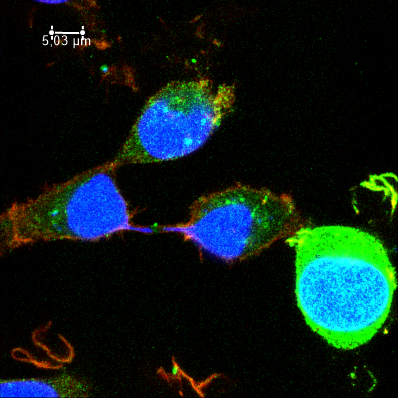

Supplement: Supplementary file 25 — Source data Fig. 3 [file 44318_2025_565_MOESM25_ESM.zip › Figure 3/3B/3B_microscopy.tif]

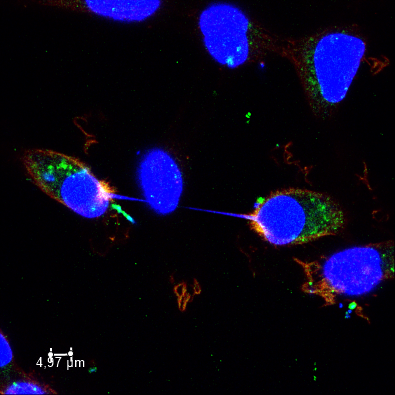

Supplement: Supplementary file 25 — Source data Fig. 3 [file 44318_2025_565_MOESM25_ESM.zip › Figure 3/3C/3C_microscopy.tif]

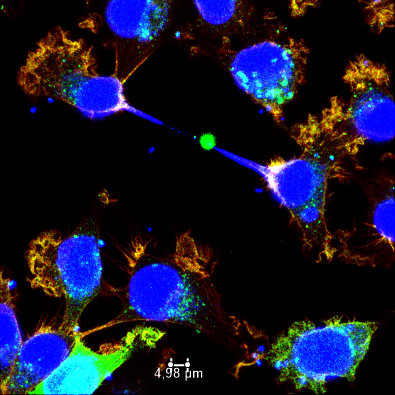

Supplement: Supplementary file 25 — Source data Fig. 3 [file 44318_2025_565_MOESM25_ESM.zip › Figure 3/3D/3D_microscopy.tif]

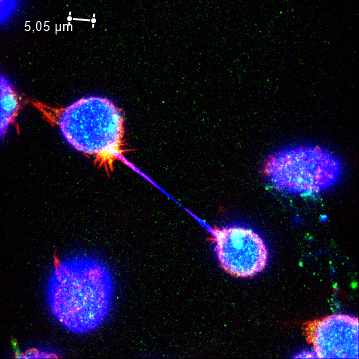

Supplement: Supplementary file 25 — Source data Fig. 3 [file 44318_2025_565_MOESM25_ESM.zip › Figure 3/3G/3G_microscopy.tif]

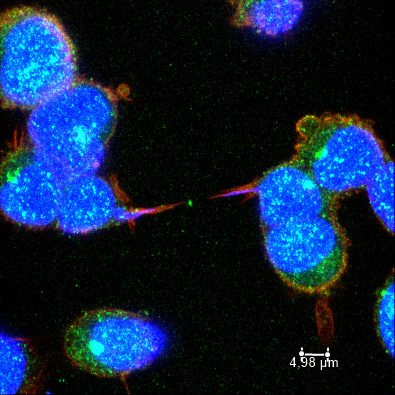

Supplement: Supplementary file 25 — Source data Fig. 3 [file 44318_2025_565_MOESM25_ESM.zip › Figure 3/3H/3H_microscopy.tif]

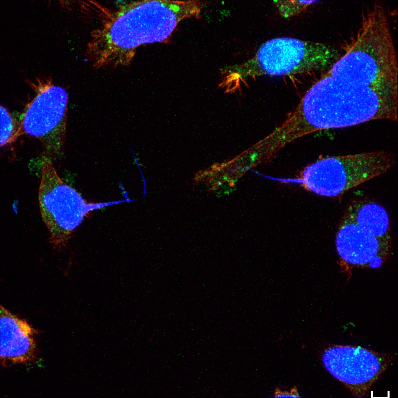

Supplement: Supplementary file 25 — Source data Fig. 3 [file 44318_2025_565_MOESM25_ESM.zip › Figure 3/3I/3I_microscopy.tif]

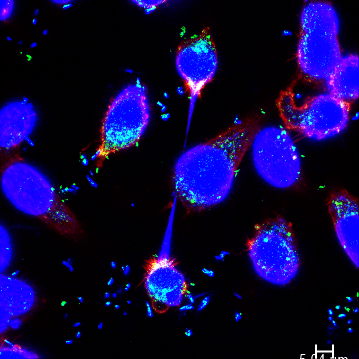

Supplement: Supplementary file 25 — Source data Fig. 3 [file 44318_2025_565_MOESM25_ESM.zip › Figure 3/3K/3K_microscopy.tif]

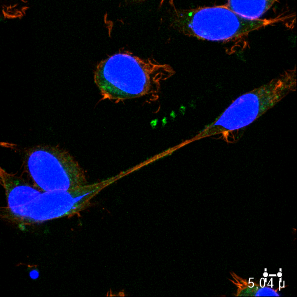

Supplement: Supplementary file 25 — Source data Fig. 3 [file 44318_2025_565_MOESM25_ESM.zip › Figure 3/3L/3L_microscopy.tif]

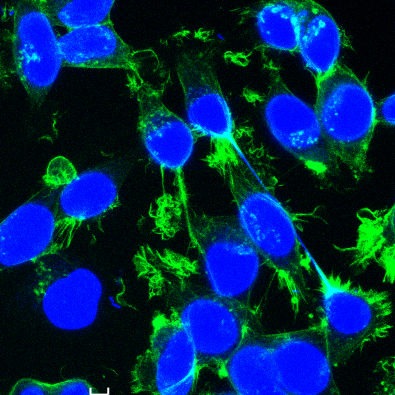

Supplement: Supplementary file 25 — Source data Fig. 3 [file 44318_2025_565_MOESM25_ESM.zip › Figure 3/3N/control/3N_microscopy_control.tif]

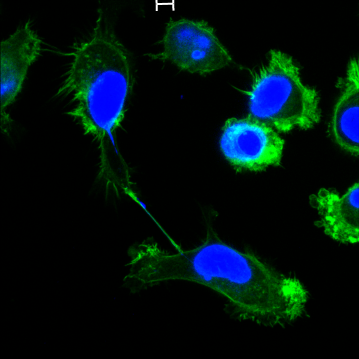

Supplement: Supplementary file 25 — Source data Fig. 3 [file 44318_2025_565_MOESM25_ESM.zip › Figure 3/3N/mDia1i/3N_microscopy_mDia1.tif]

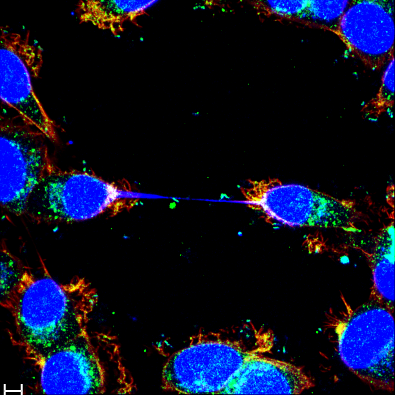

Supplement: Supplementary file 26 — Source data Fig. 4 [file 44318_2025_565_MOESM26_ESM.zip › Figure 4/4C/4C_microscopy.tif]

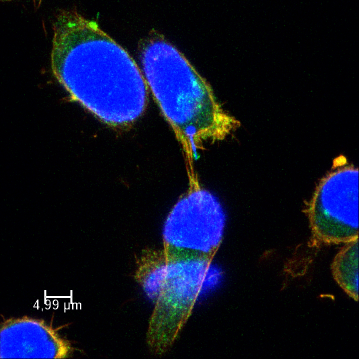

Supplement: Supplementary file 26 — Source data Fig. 4 [file 44318_2025_565_MOESM26_ESM.zip › Figure 4/4D/4D_microscopy.tif]

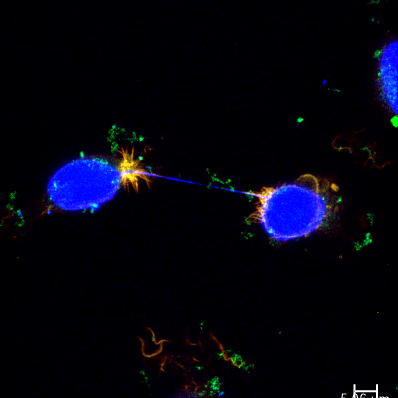

Supplement: Supplementary file 26 — Source data Fig. 4 [file 44318_2025_565_MOESM26_ESM.zip › Figure 4/4F/4F_microscopy.tif]

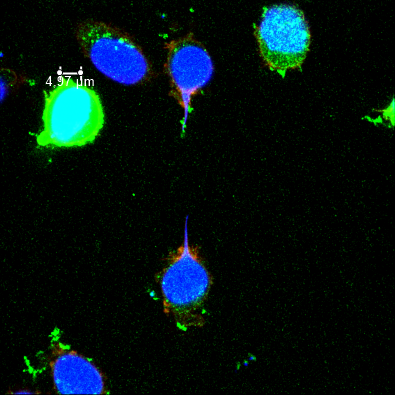

Supplement: Supplementary file 26 — Source data Fig. 4 [file 44318_2025_565_MOESM26_ESM.zip › Figure 4/4G/4G_microscopy.tif]

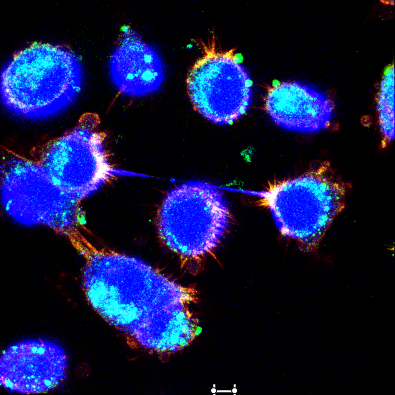

Supplement: Supplementary file 26 — Source data Fig. 4 [file 44318_2025_565_MOESM26_ESM.zip › Figure 4/4H/4H_microscopy.tif]

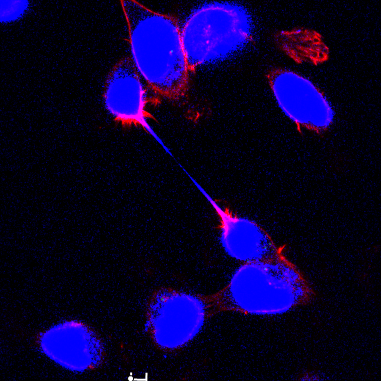

Supplement: Supplementary file 26 — Source data Fig. 4 [file 44318_2025_565_MOESM26_ESM.zip › Figure 4/4O/4O_microscopy.tif]

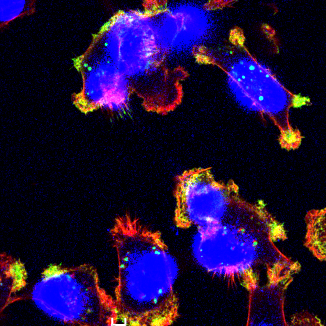

Supplement: Supplementary file 26 — Source data Fig. 4 [file 44318_2025_565_MOESM26_ESM.zip › Figure 4/4P/4P_microscopy.tif]

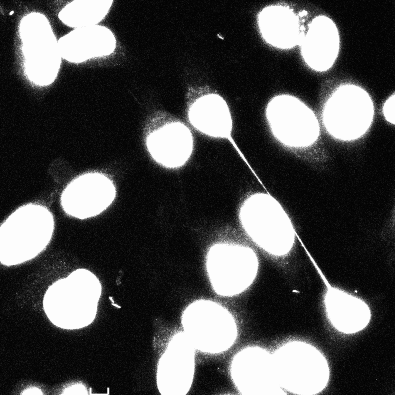

Supplement: Supplementary file 27 — Source data Fig. 5 [file 44318_2025_565_MOESM27_ESM.zip › Figure 5/5F/5F_microscopy.tif]

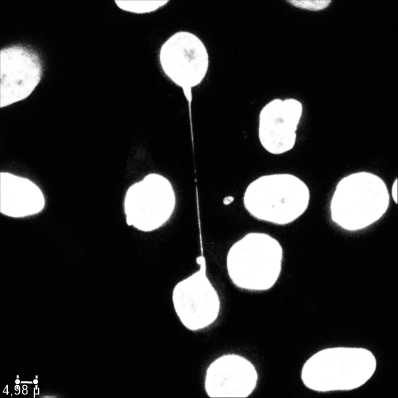

Supplement: Supplementary file 27 — Source data Fig. 5 [file 44318_2025_565_MOESM27_ESM.zip › Figure 5/5G/5G_microscopy.tif]

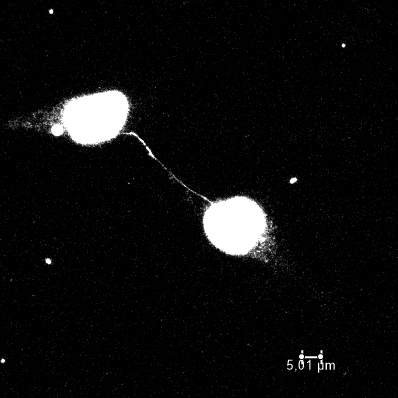

Supplement: Supplementary file 27 — Source data Fig. 5 [file 44318_2025_565_MOESM27_ESM.zip › Figure 5/5H/5H_microscopy.tif]

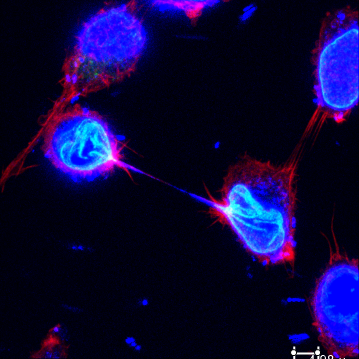

Supplement: Supplementary file 27 — Source data Fig. 5 [file 44318_2025_565_MOESM27_ESM.zip › Figure 5/5K/5K_microscopy.tif]

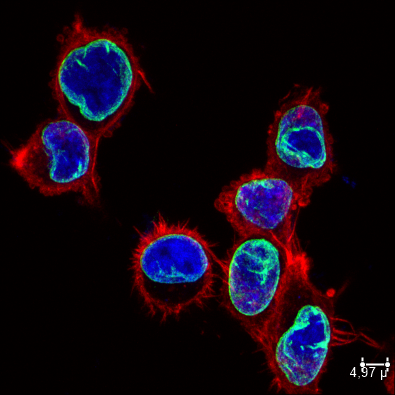

Supplement: Supplementary file 27 — Source data Fig. 5 [file 44318_2025_565_MOESM27_ESM.zip › Figure 5/5L/5L_microscopy.tif]

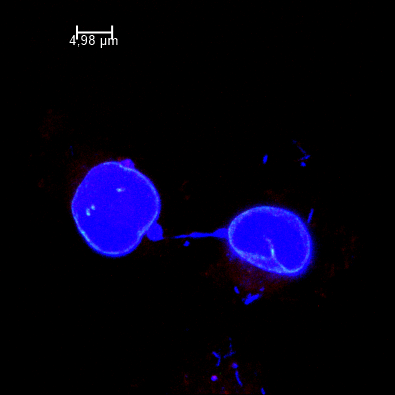

Supplement: Supplementary file 27 — Source data Fig. 5 [file 44318_2025_565_MOESM27_ESM.zip › Figure 5/5M/5M_microscopy.tif]

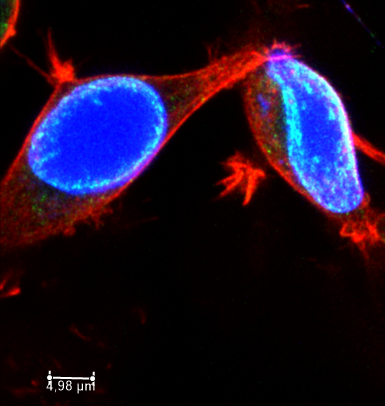

Supplement: Supplementary file 27 — Source data Fig. 5 [file 44318_2025_565_MOESM27_ESM.zip › Figure 5/5Q/5Q_microscopy.tif]

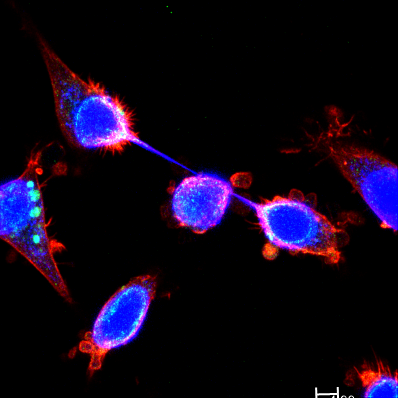

Supplement: Supplementary file 27 — Source data Fig. 5 [file 44318_2025_565_MOESM27_ESM.zip › Figure 5/5R/5R_microscopy.tif]

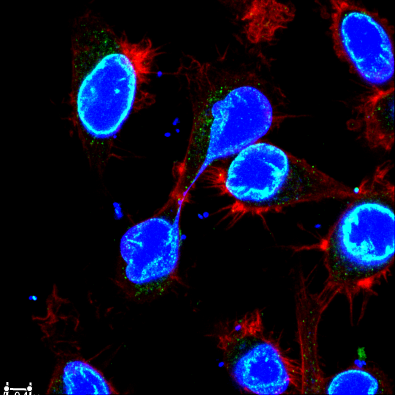

Supplement: Supplementary file 27 — Source data Fig. 5 [file 44318_2025_565_MOESM27_ESM.zip › Figure 5/5U/5U_microscopy.tif]

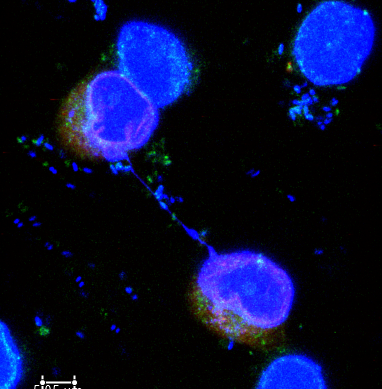

Supplement: Supplementary file 27 — Source data Fig. 5 [file 44318_2025_565_MOESM27_ESM.zip › Figure 5/5V/5V_microscopy.tif]

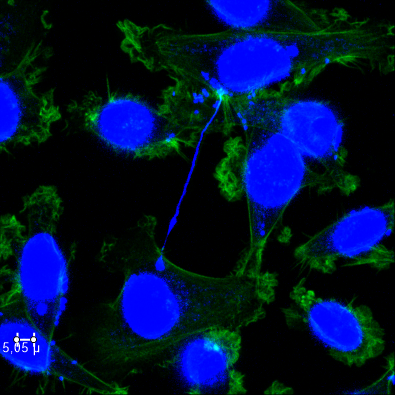

Supplement: Supplementary file 27 — Source data Fig. 5 [file 44318_2025_565_MOESM27_ESM.zip › Figure 5/5W/5W_microscopy.tif]

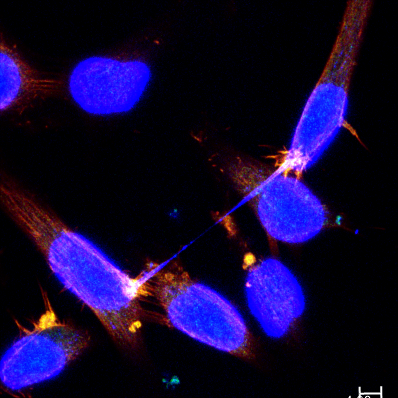

Supplement: Supplementary file 28 — Source data Fig. 6 [file 44318_2025_565_MOESM28_ESM.zip › Figure 6/6A/6A_microscopy.tif]

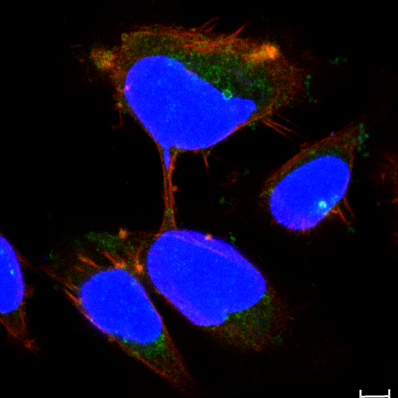

Supplement: Supplementary file 28 — Source data Fig. 6 [file 44318_2025_565_MOESM28_ESM.zip › Figure 6/6B/6B_microscopy.tif]

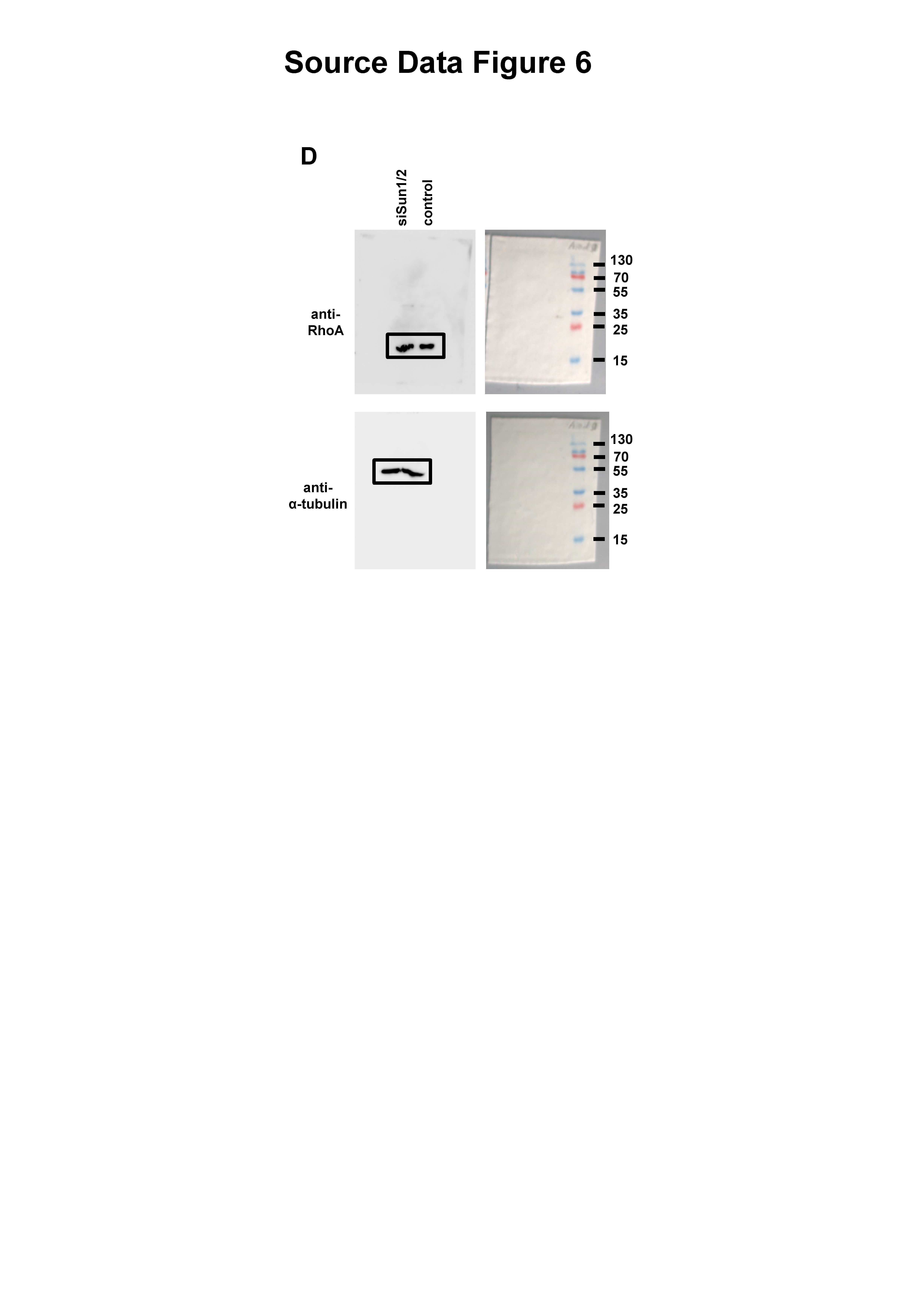

Supplement: Supplementary file 28 — Source data Fig. 6 [file 44318_2025_565_MOESM28_ESM.zip › Figure 6/6D/6D.tif]

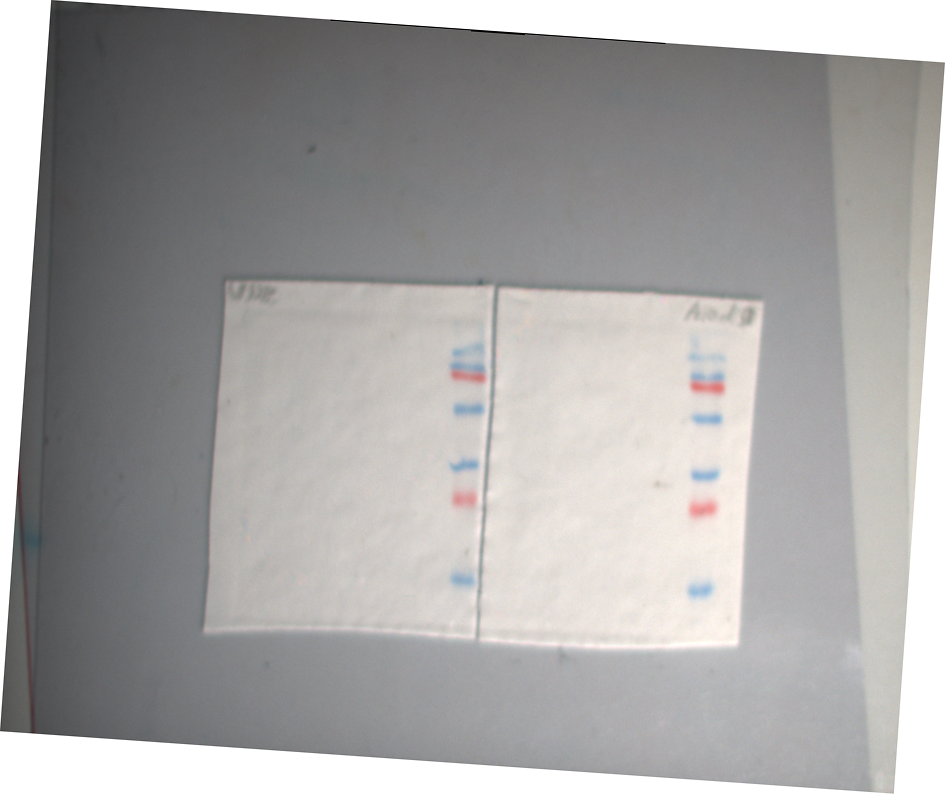

Supplement: Supplementary file 28 — Source data Fig. 6 [file 44318_2025_565_MOESM28_ESM.zip › Figure 6/6D/RhoA/RhoA_marker.tif]

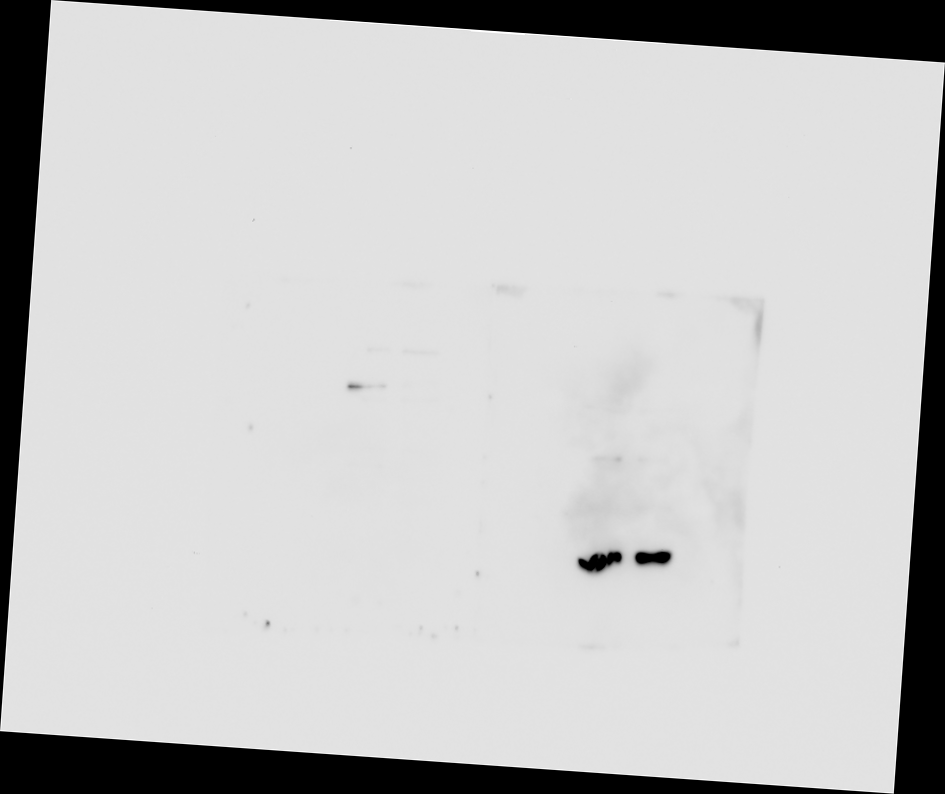

Supplement: Supplementary file 28 — Source data Fig. 6 [file 44318_2025_565_MOESM28_ESM.zip › Figure 6/6D/RhoA/RhoA_western.tif]

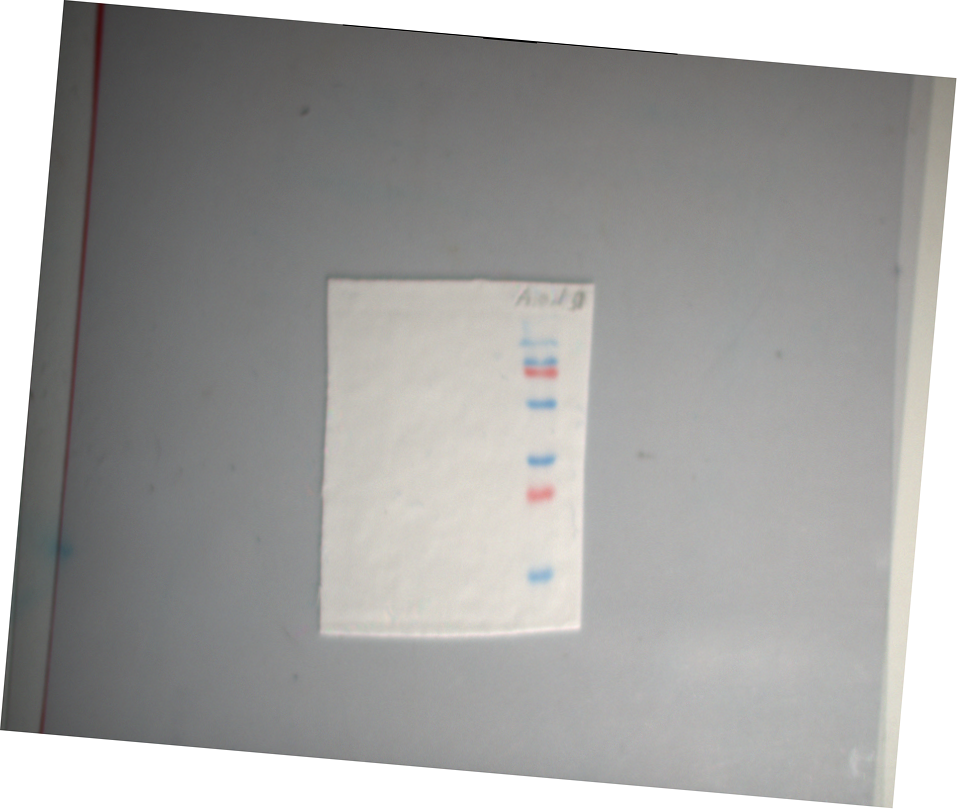

Supplement: Supplementary file 28 — Source data Fig. 6 [file 44318_2025_565_MOESM28_ESM.zip › Figure 6/6D/α-tubulin/a-tubulin_marker.tif]

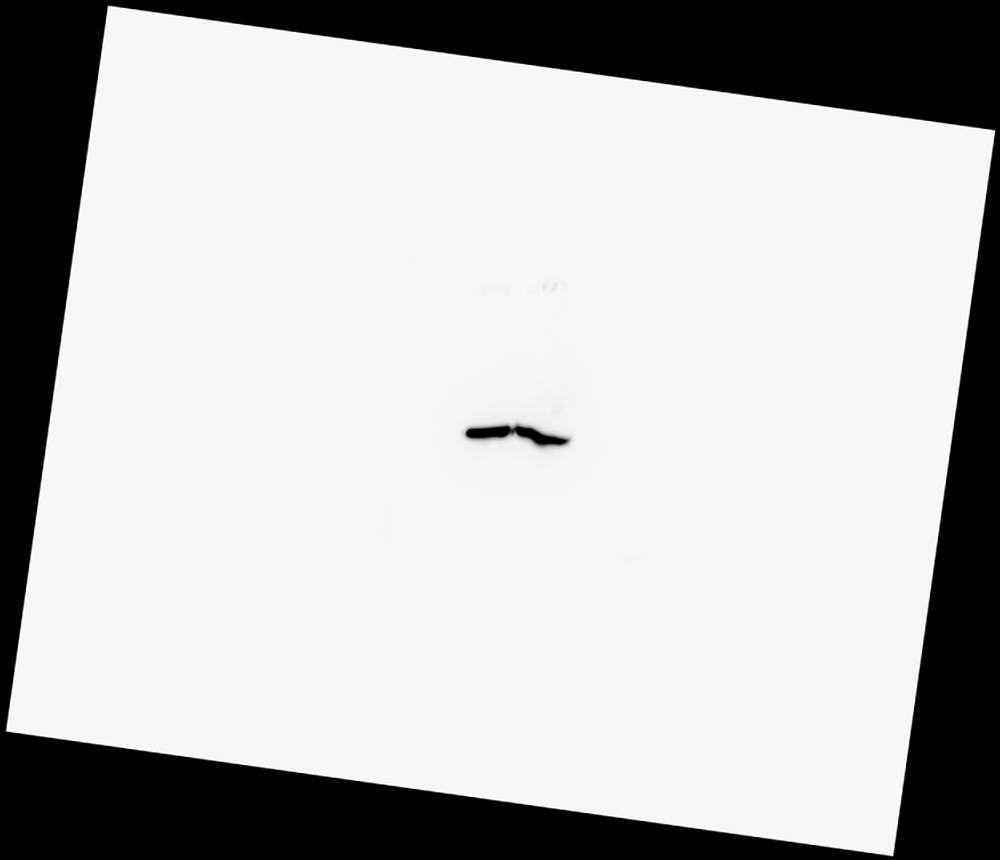

Supplement: Supplementary file 28 — Source data Fig. 6 [file 44318_2025_565_MOESM28_ESM.zip › Figure 6/6D/α-tubulin/a-tubulin_western.tif]

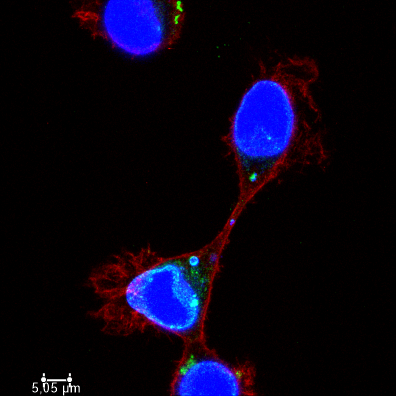

Supplement: Supplementary file 28 — Source data Fig. 6 [file 44318_2025_565_MOESM28_ESM.zip › Figure 6/6K/6K_microscopy.tif]

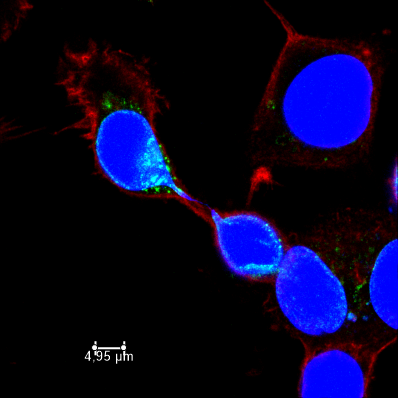

Supplement: Supplementary file 28 — Source data Fig. 6 [file 44318_2025_565_MOESM28_ESM.zip › Figure 6/6N/6N_microscopy.tif]

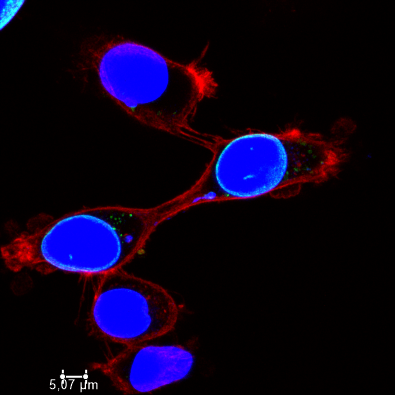

Supplement: Supplementary file 28 — Source data Fig. 6 [file 44318_2025_565_MOESM28_ESM.zip › Figure 6/6O/6O_microscopy.tif]

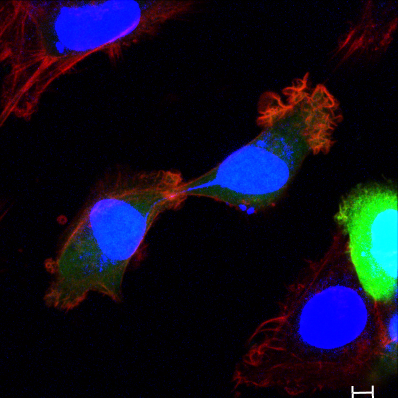

Supplement: Supplementary file 29 — Source data Fig. 7 [file 44318_2025_565_MOESM29_ESM.zip › Figure 7/7A/7A_microscopy.tif]

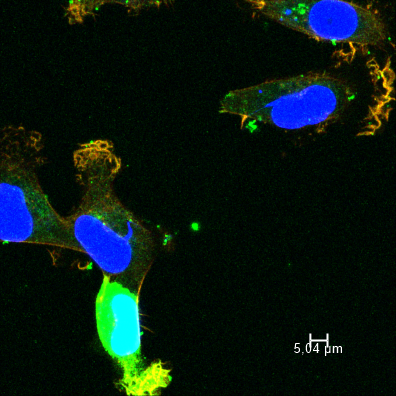

Supplement: Supplementary file 29 — Source data Fig. 7 [file 44318_2025_565_MOESM29_ESM.zip › Figure 7/7B/7B_microscopy.tif]

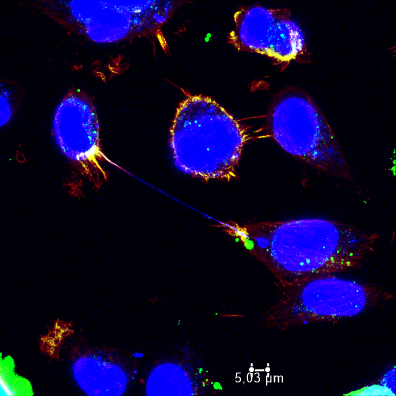

Supplement: Supplementary file 29 — Source data Fig. 7 [file 44318_2025_565_MOESM29_ESM.zip › Figure 7/7D/7D_microscopy.tif]

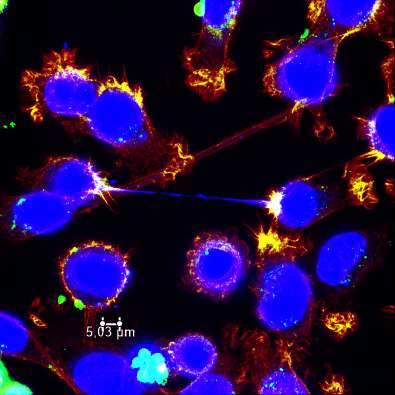

Supplement: Supplementary file 29 — Source data Fig. 7 [file 44318_2025_565_MOESM29_ESM.zip › Figure 7/7E/7E_microscopy.tif]

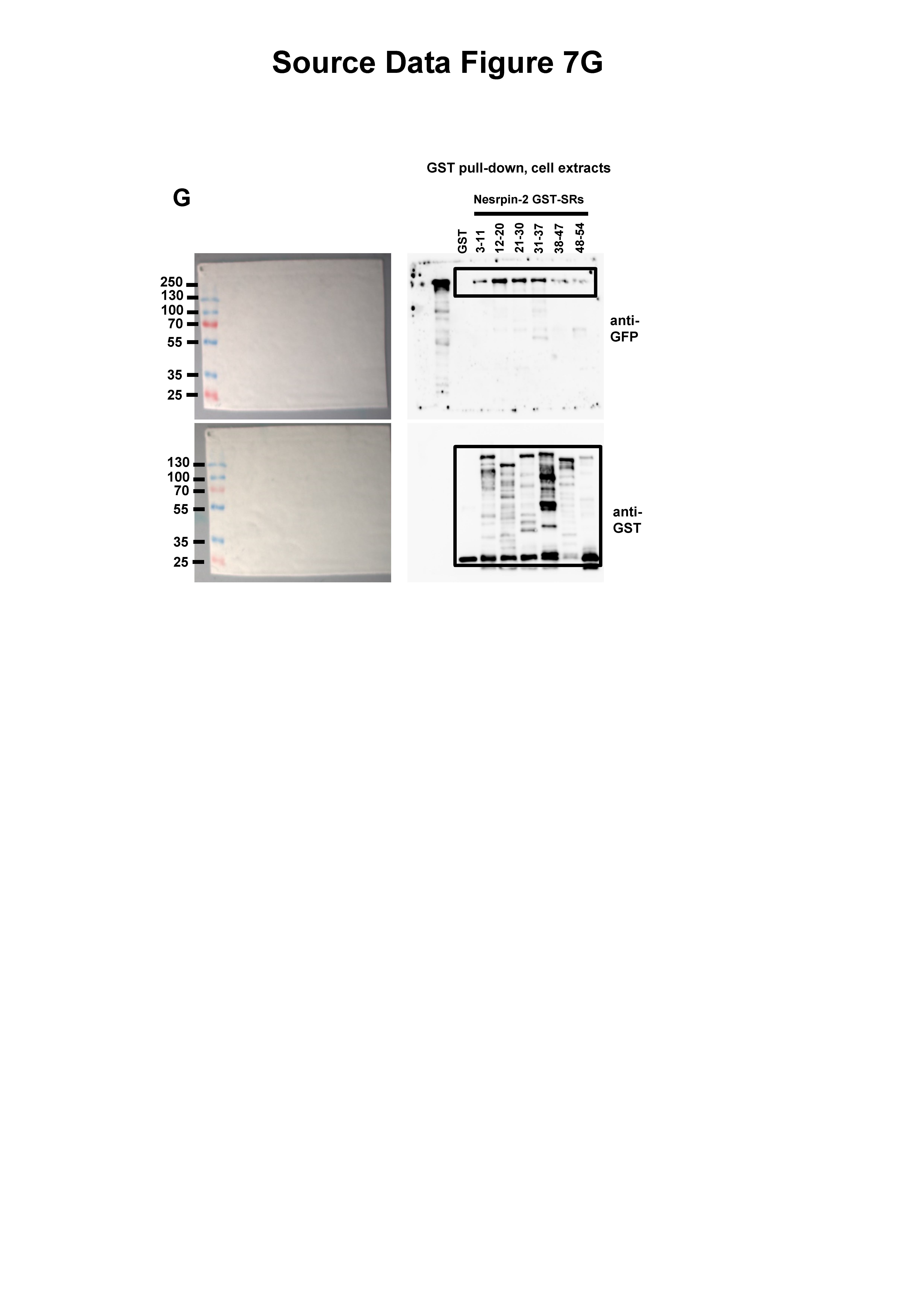

Supplement: Supplementary file 29 — Source data Fig. 7 [file 44318_2025_565_MOESM29_ESM.zip › Figure 7/7G/7G.tif]

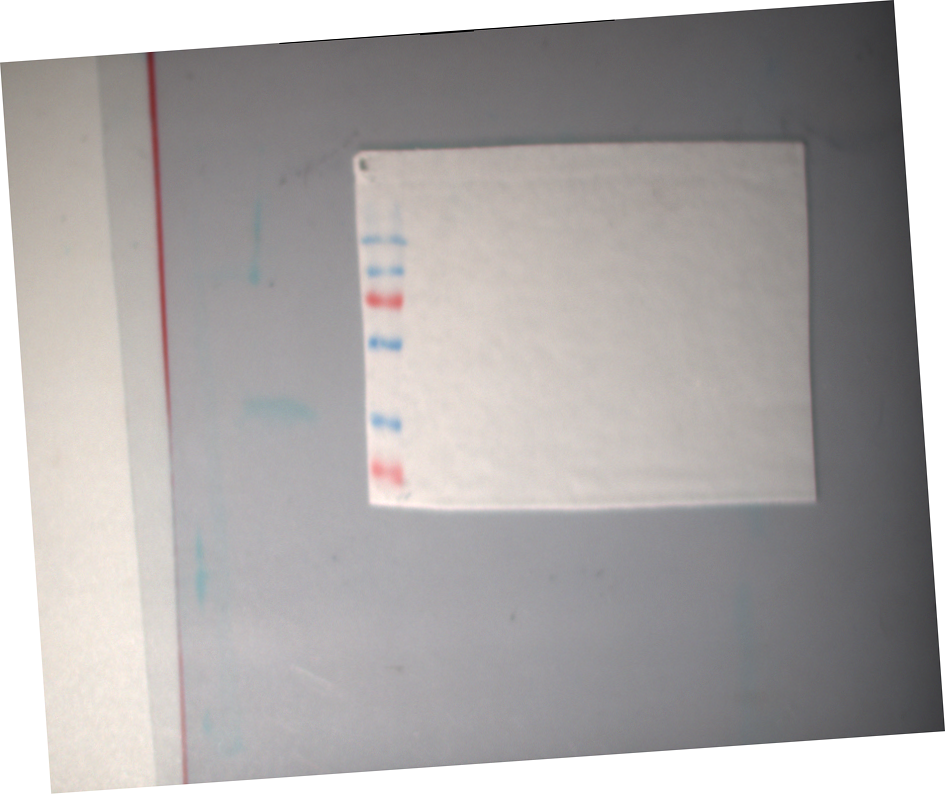

Supplement: Supplementary file 29 — Source data Fig. 7 [file 44318_2025_565_MOESM29_ESM.zip › Figure 7/7G/GFP (Citrine)/GFP_marker.tif]

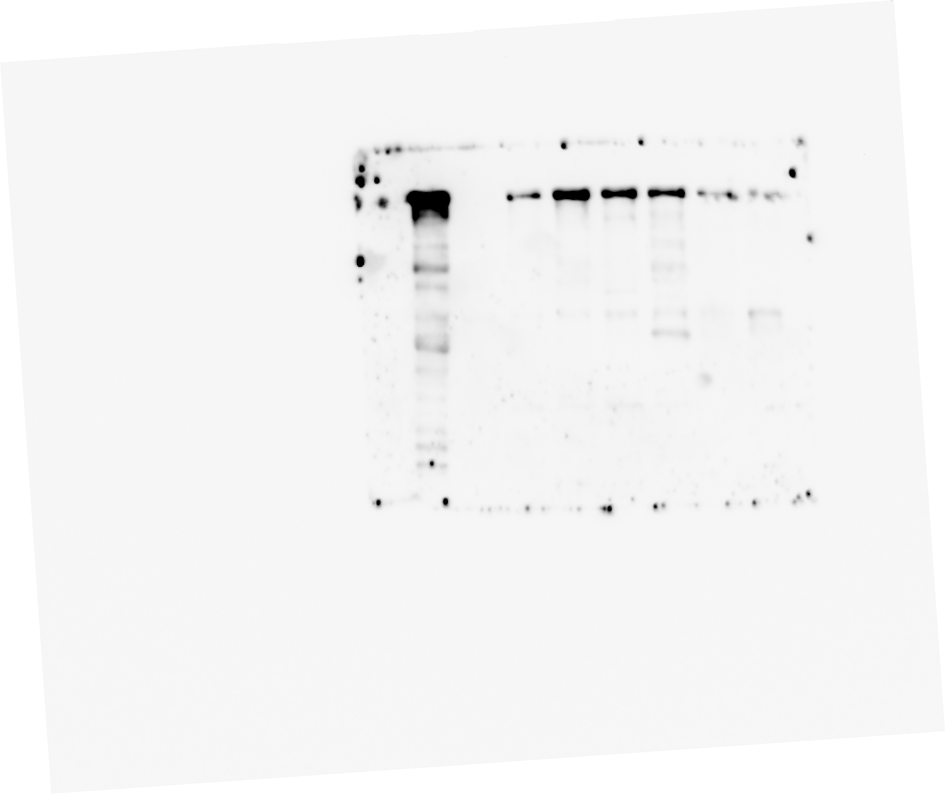

Supplement: Supplementary file 29 — Source data Fig. 7 [file 44318_2025_565_MOESM29_ESM.zip › Figure 7/7G/GFP (Citrine)/GFP_western.tif]

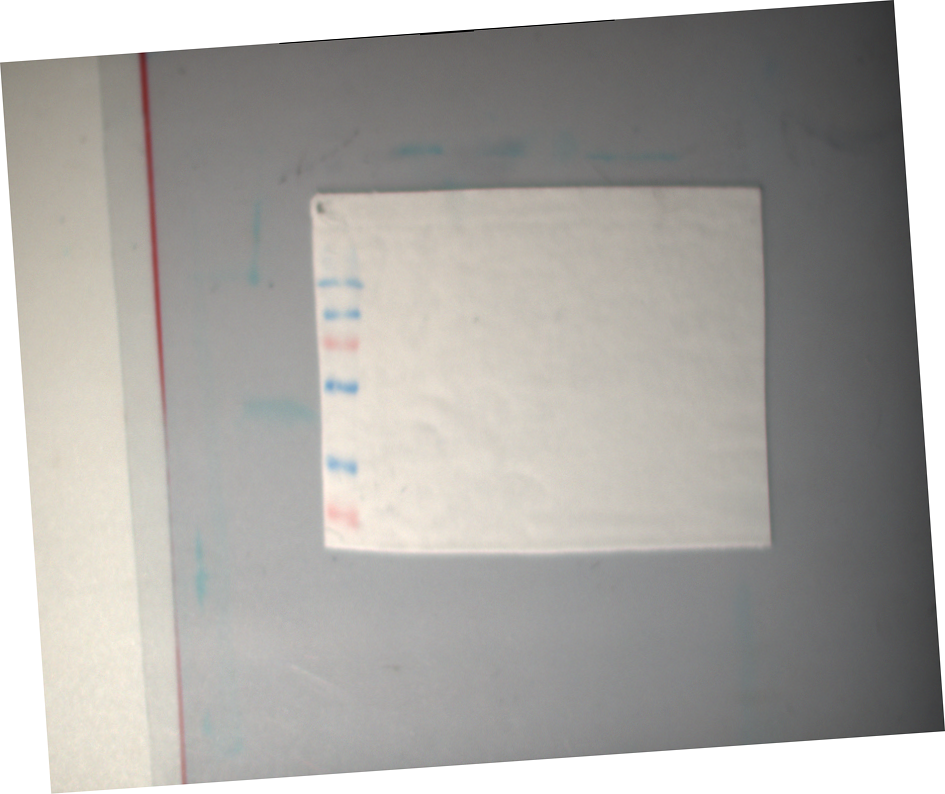

Supplement: Supplementary file 29 — Source data Fig. 7 [file 44318_2025_565_MOESM29_ESM.zip › Figure 7/7G/GST/GST_marker.tif]

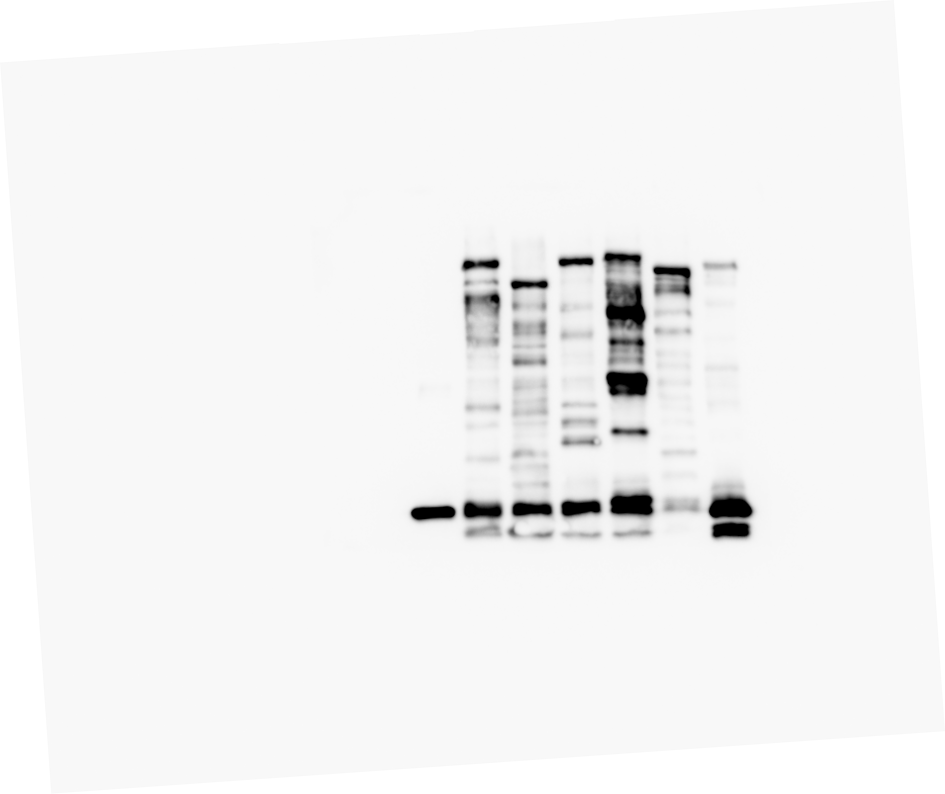

Supplement: Supplementary file 29 — Source data Fig. 7 [file 44318_2025_565_MOESM29_ESM.zip › Figure 7/7G/GST/GST_western.tif]

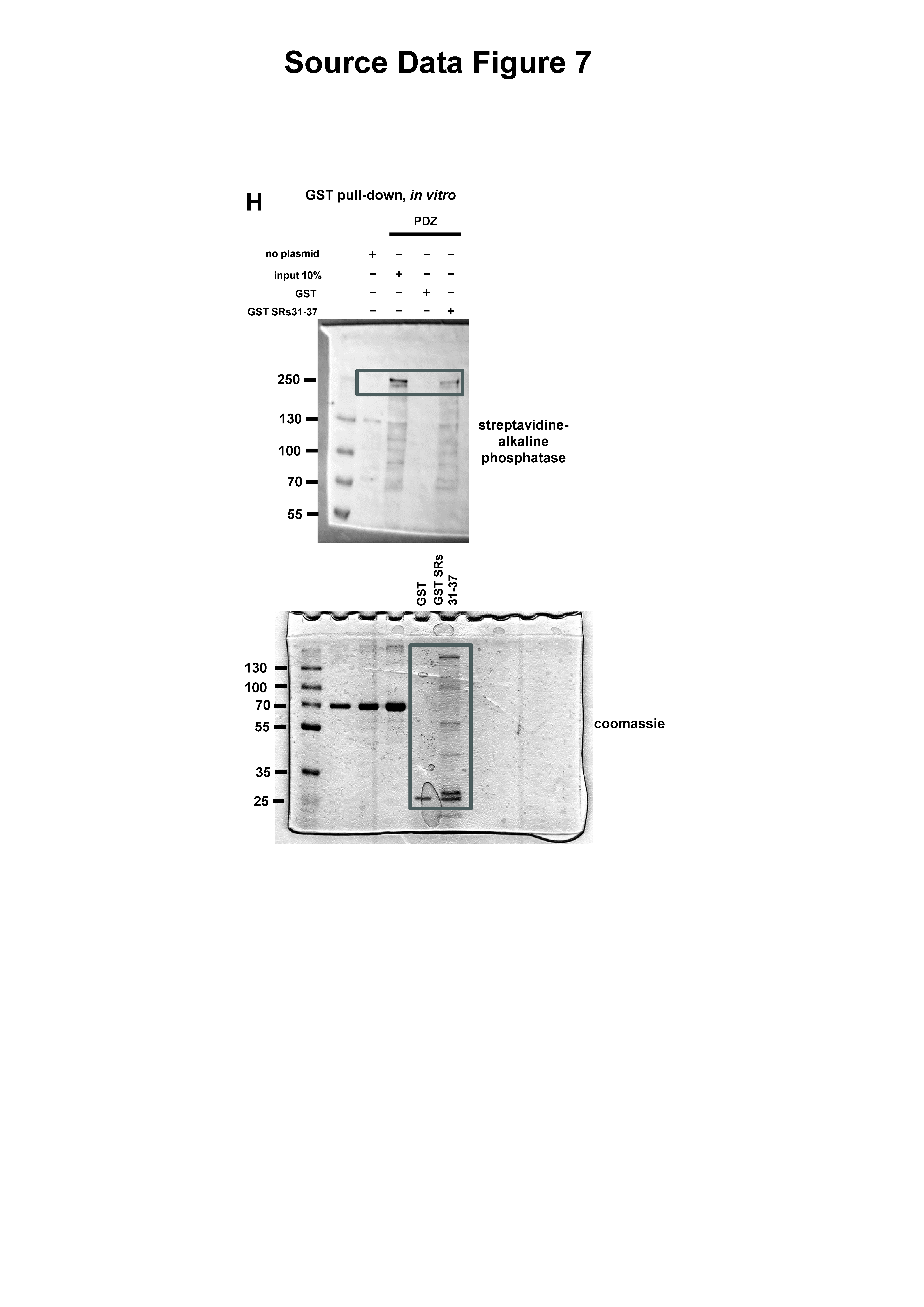

Supplement: Supplementary file 29 — Source data Fig. 7 [file 44318_2025_565_MOESM29_ESM.zip › Figure 7/7H/7H.tif]

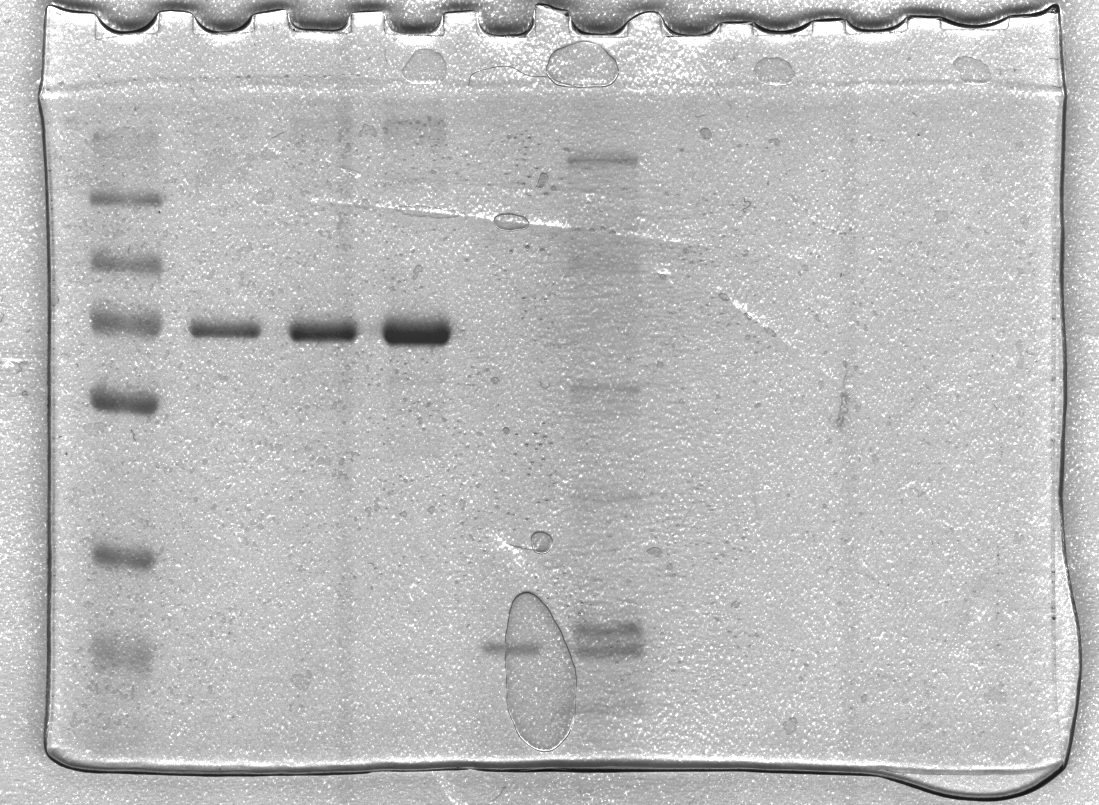

Supplement: Supplementary file 29 — Source data Fig. 7 [file 44318_2025_565_MOESM29_ESM.zip › Figure 7/7H/GST_coomassie.tif]

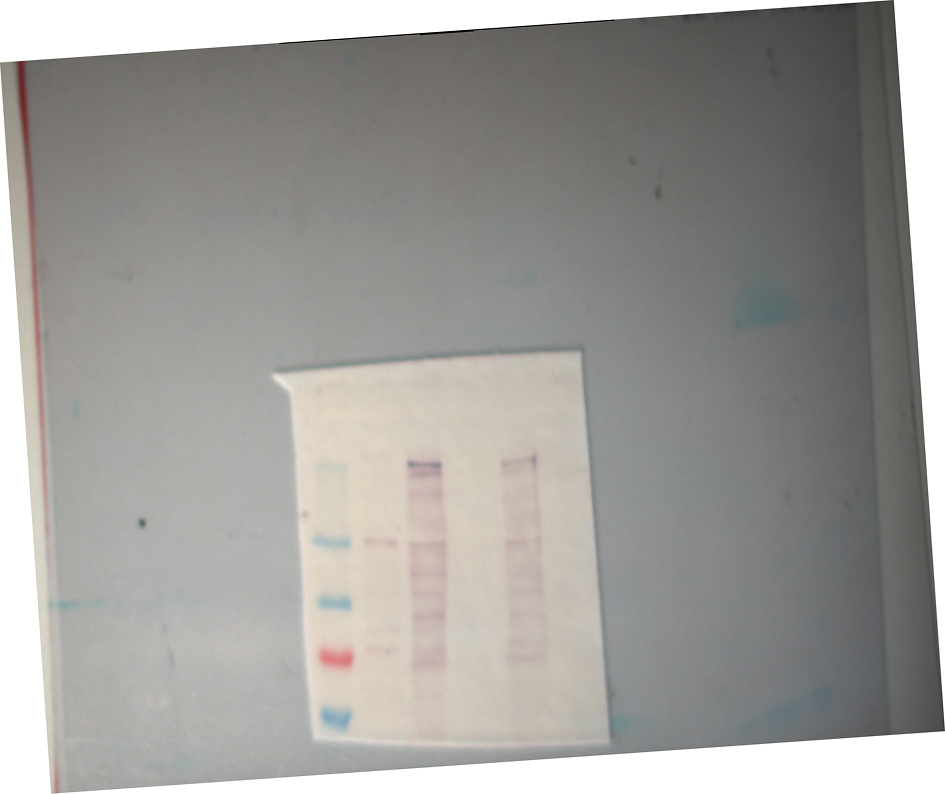

Supplement: Supplementary file 29 — Source data Fig. 7 [file 44318_2025_565_MOESM29_ESM.zip › Figure 7/7H/PDZ_alkaline phosphatase.tif]

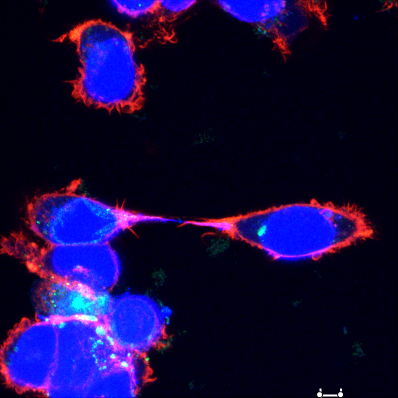

Supplement: Supplementary file 29 — Source data Fig. 7 [file 44318_2025_565_MOESM29_ESM.zip › Figure 7/7L/7L_microscopy.tif]

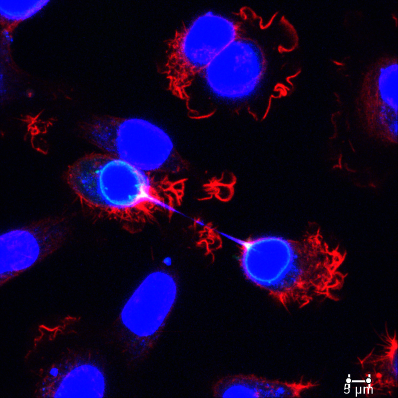

Supplement: Supplementary file 29 — Source data Fig. 7 [file 44318_2025_565_MOESM29_ESM.zip › Figure 7/7M/7M_microscopy.tif]

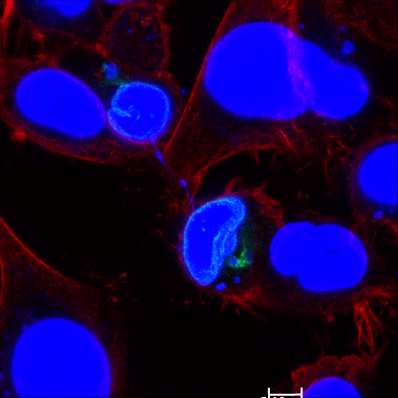

Supplement: Supplementary file 29 — Source data Fig. 7 [file 44318_2025_565_MOESM29_ESM.zip › Figure 7/7N/7N_microscopy.tif]

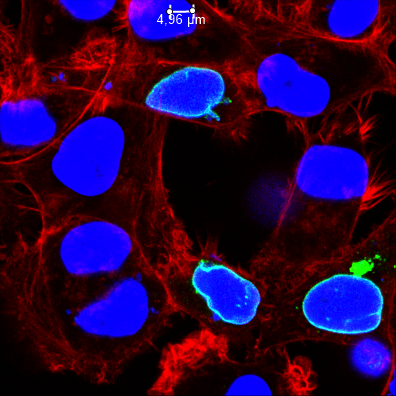

Supplement: Supplementary file 29 — Source data Fig. 7 [file 44318_2025_565_MOESM29_ESM.zip › Figure 7/7O/7O_microscopy.tif]

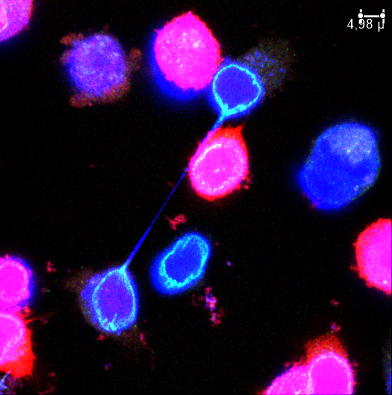

Supplement: Supplementary file 30 — Source data Fig. 8 [file 44318_2025_565_MOESM30_ESM.zip › Figure 8/8B/8B_microscopy.tif]
