# Supplementary figures and images for: Tension-sensitive LINC-RhoA signaling prevents chromatin bridge breakage in cytokinesis (part 2 of 2)
Source: EMBO J. 2025 Sep 9;44(20):5834–59. doi: 10.1038/s44318-025-00565-3 (PMC12528419; doi:10.1038/s44318-025-00565-3)

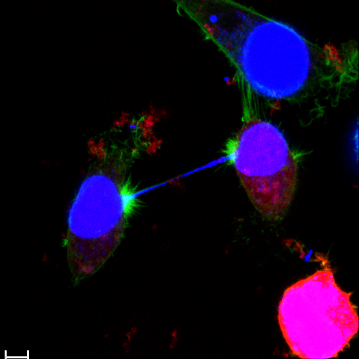

Supplement: Supplementary file 30 — Source data Fig. 8 [file 44318_2025_565_MOESM30_ESM.zip › Figure 8/8C/8C_microscopy.tif]

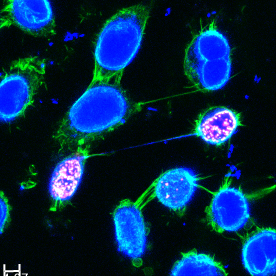

Supplement: Supplementary file 30 — Source data Fig. 8 [file 44318_2025_565_MOESM30_ESM.zip › Figure 8/8D/8D_microscopy.tif]

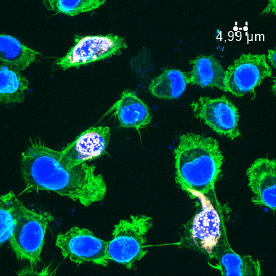

Supplement: Supplementary file 30 — Source data Fig. 8 [file 44318_2025_565_MOESM30_ESM.zip › Figure 8/8E/8E_microscopy.tif]

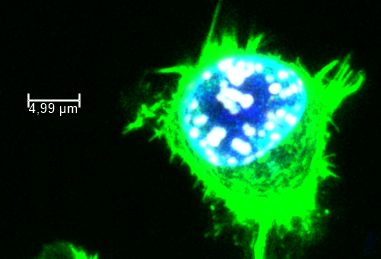

Supplement: Supplementary file 30 — Source data Fig. 8 [file 44318_2025_565_MOESM30_ESM.zip › Figure 8/8L/8L_microscopy.tif]

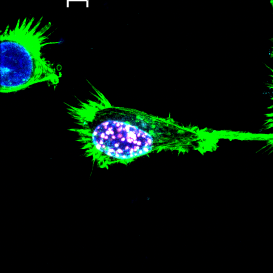

Supplement: Supplementary file 30 — Source data Fig. 8 [file 44318_2025_565_MOESM30_ESM.zip › Figure 8/8M/8M_microscopy.tif]

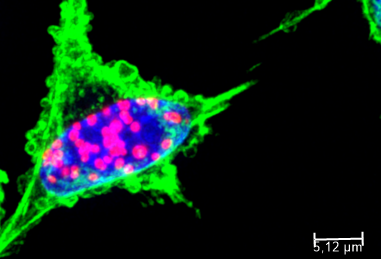

Supplement: Supplementary file 30 — Source data Fig. 8 [file 44318_2025_565_MOESM30_ESM.zip › Figure 8/8N/8N_microscopy.tif]
